# HUWE1 in Skeletal Muscle Prevents Muscle Fatigue via Maintaining Iron and Calcium Homeostasis

**DOI:** 10.1002/advs.202516719

**Published:** 2025-11-20

**Authors:** Huike Jiao, Yuting Du, Danxia Zhou, Meng Bai, Xu Gao, Zhenyue Hao, Nan‐Jie Xu, Jiaqiang Liu, Ying Huang, Zhenji Gan, Jing Zhang

**Affiliations:** ^1^ Institute for Translational Medicine on Cell Fate and Disease Shanghai Ninth People's Hospital Key Laboratory of Cell Differentiation and Apoptosis of National Ministry of Education Department of Pathophysiology Shanghai Jiao Tong University School of Medicine Shanghai 200025 China; ^2^ Key Laboratory of Cell Differentiation and Apoptosis of National Ministry of Education Department of Pathophysiology Shanghai Jiao Tong University School of Medicine Shanghai 200025 China; ^3^ The State Key Laboratory of Pharmaceutical Biotechnology and MOE Key Laboratory of Model Animal for Disease Study Model Animal Research Center Nanjing University Medical School Nanjing University Nanjing 210000 China; ^4^ Princess Margaret Cancer Centre University Health Network Toronto ON M5G 2C1 Canada; ^5^ Songjiang Hospital and Songjiang Research Institute Shanghai Key Laboratory of Emotions and Affective Disorders Shanghai Jiao Tong University School of Medicine Shanghai 201600 China; ^6^ Department of Oral and Cranio‐maxillofacial Surgery Shanghai Ninth People's Hospital Shanghai Jiao Tong University School of Medicine Shanghai 200011 China

**Keywords:** Exercise, HUWE1, Iron overload, Oxidative stress, SERCA

## Abstract

Iron is critical to optimal athletic performance because of its role in energy metabolism, oxygen transport, and acid‐base balance. However, the precise mechanism how skeletal muscle maintains iron homeostasis during exercise remains enigmatic. Here, it is demonstrated that the HECT‐domain containing ubiquitin ligase E3 Huwe1 (also known as MULE or ARF‐BP1) in skeletal muscle is suppressed upon exhausted exercise. Loss of Huwe1 in skeletal muscle restrains the exercise performance of Huwe1 conditional knockout (cKO) mice, accompanied with pronounced oxidative stress. Mechanistically, Huwe1 depletion stabilizes c‐Myc protein, leading to upregulated sarcolipin (Sln) expression with inhibited SarcoEndoplasmic Reticulum Calcium ATPase (SERCA) activity, and downregulated ferroportin (Fpn, also known as Slc40a1) expression with iron overload. Silencing of c‐Myc restores SERCA activity and iron export. Consistently, SERCA activator CDN1163, Sln silencing, or dietary iron restriction ameliorates the exercise performance of *Huwe1* cKO mice. Of note, improved exercise performance is accompanied with diminished oxidative stress in *Huwe1* cKO mice upon iron restriction. Taken together, the results unveil a key function for HUWE1 in skeletal muscle as a fundamental coordinator of iron and calcium homeostasis by regulating SERCA activity and iron metabolism. These findings reveal a regulatory pathway on controlling iron/calcium homeostasis and exercise capacity.

## Graphical abstract

This study investigates the impact of iron homeostasis on exercise. Loss of the HECT domain‐containing ubiquitin ligase E3 (HUWE1) in skeletal muscle restrains the exercise performance in mice with downregulated ferroportin expression leading to iron overload, which is ameliorated by dietary iron restriction. Therefore, it unveils a key function for HUWE1 in skeletal muscle to coordinate iron homeostasis during exercise.

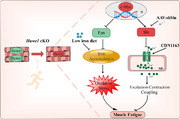

## 1 Introduction

Substantial evidence has suggested that regular physical activity of moderate intensity daily is beneficial for the maintenance of overall health and reductions in potential disease risks.^[^ ^1^ ^]^ However, it is well‐recognized that unaccustomed or exhausted exercise results in detrimental health effects and induces oxidative stress.^[^ ^2^ ^]^ Although it's still under debate about the primary tissue responsible for the production of reactive oxygen species (ROS) during exercise, compelling evidence indicates that contracting muscle fibers are a primary source of ROS production during exercise. The generated ROS not only contributes to increased protein oxidation and lipid peroxidation but also accelerates muscle fatigue as a result.^[^ ^3^, ^4^ ^]^

Fe^2+^ is an important metal ion for muscle function, which is a potential factor contributing to muscle oxidative stress. Ferroportin (FPN, encoded by SLC40A1) is the only mammalian nonheme cellular iron exporter identified to date, which plays an essential role in transporting iron from intracellular to extracellular environments.^[^ ^5^, ^6^, ^7^ ^]^ When FPN expression is abnormal, cellular iron metabolism becomes dysregulated, resulting in an overload of intracellular iron.^[^ ^8^ ^]^ In fact, a high amount of iron has been detected in the skeletal muscle, in which the total amount of stored iron is comparable with that in the liver.^[^ ^9^ ^]^ Evidence has implicated that iron overload‐induced oxidative stress can negatively affect the function of skeletal muscle.^[^ ^10^ ^]^ However, so far, how iron metabolism contributes to oxidative stress in contracting muscles has not been clearly elucidated.

In addition to iron, skeletal muscle contraction relies on high‐fidelity calcium (Ca^2+^) signals. Sarcoendoplasmic reticulum (SR) is the primary storage site for calcium, which is mobilized by calcium release channels for the activation of the contractile machinery within the cytosol (systole). Following muscle contraction, most of the cytosolic calcium is recovered into the SR to initiate muscle relaxation (diastole). The release and recovery of calcium into the SR lumen ensures cells can begin the contraction‐relaxation cycle anew.^[^ ^11^, ^12^ ^]^ As a key regulator of cellular calcium homeostasis, the SarcoEndoplasmic Reticulum Calcium ATPase (SERCA) pump acts to transport calcium ions from the cytosol back to the SR following muscle contraction. The Ca^2+^ sequestering activity of SERCA is inhibited by the endogenous proteins phospholamban (PLN) and sarcolipin (SLN).^[^ ^13^ ^]^ Depressed SERCA function is associated with impairment of intracellular calcium homeostasis and further contributes to muscle fatigue.^[^ ^14^ ^]^ On the contrary, restoration of SERCA ATPase prevents oxidative stress‐related muscle weakness.^[^ ^15^ ^]^

HUWE1 (also known as MULE or ARF‐BP1), a giant HECT‐domain E3 ubiquitin ligase,^[^ ^16^ ^]^ modulates a wide array of biological processes such as cell growth, DNA repair, cell death, and stress response through diverse substrates, including MCL‐1, P53, c‐MYC, and so on.^[^ ^17^, ^18^, ^19^, ^20^, ^21^, ^22^ ^]^ Our previous work suggested that HUWE1 functions as a novel protector against oxidative stress in acute liver injury.^[^ ^22^ ^]^ Specifically, loss of HUWE1 impedes the ubiquitination and degradation of transferrin receptor (TfR1), leading to aberrant iron accumulation and thus lipid peroxidation.^[^ ^22^ ^]^ So far, little is known about the role of HUWE1 in oxidative stress of various physiological conditions, such as exhaustive exercise.

In this study, we reported that Huwe1 was downregulated upon exhausted exercise. Mice with loss‐of‐function of Huwe1 in skeletal muscle (*Huwe1* cKO mice) displayed weakened exercise capacity compared to wild‐type (WT) mice. This was achieved by increased Sln expression to suppress SERCA activity and decreased Fpn expression to potentiate iron accumulation, which contributes to oxidative stress via stabilized c‐Myc in Huwe1 deficient mice. Consequently, SERCA allosteric activator CDN1163, knockdown of Sln, and dietary iron restriction all improved the exercise weakness of *Huwe1* cKO mice. Of note, improved exercise performance was accompanied with diminished oxidative stress in *Huwe1* cKO mice upon iron restriction. Collectively, these studies indicate that Huwe1 in skeletal muscle is a crucial coordinator of iron and calcium homeostasis via regulating Fpn/iron and Sln/SERCA. These findings highlight a novel mechanism through which HUWE1 acts as a modulator of iron and calcium homeostasis in exercise of skeletal muscle.

## 2 Results

### 2.1 Huwe1 Expression is Suppressed upon Exhausted Exercise in the Soleus Muscle

Given an intimate association of HUWE1 with oxidative stress, as well as the apparent functional role of oxidative stress in exercise, we hypothesized that HUWE1 may play a pivotal role in exercise. To prove this hypothesis, we first evaluated the dynamic change of HUWE1 expression in skeletal muscle upon exercise. To this end, 27 transcriptomic datasets for the muscle of healthy humans subjected to disuse or aerobic exercise were analyzed.^[^ ^23^ ^]^ The RNA levels of HUWE1 were downregulated upon acute exercise (Sedentary, Sed _mean_ = 0.19 versus Acute Exercise, AE _mean_ = 0.027, *p* = 0.0119, Figure S1A, Supporting Information), although it's challenging to compare the protein levels of HUWE1 in human biopsies upon exercise. Meanwhile, C57BL/6 wild‐type mice were subjected to exhausted exercise to test the dynamic change of Huwe1 expression in mice. Both the mRNA and protein levels of Huwe1 were significantly decreased after one bout of exhausted exercise, after 3 hours, which turned to be comparable to that in the sedentary mice (Figure S1B,C, Supporting Information). Of note, there was no obvious change on Huwe1 ubiquitination post‐exhausted exercise, suggesting the proteasome degradation system is not the major factor for the reduction of Huwe1 after exercise. (Figure S1D, Supporting Information). Moreover, Huwe1 was specifically decreased in oxidative fibers such as soleus after exercise, while remaining unchanged in glycolytic fibers such as extensor digitorum longus (EDL) muscles (Figure S1E, Supporting Information). These data suggested that Huwe1 may act as a coordinator in regulating the exercise process.

### 2.2 Mice with Loss‐of‐Function of Huwe1 in Skeletal Muscle Display Reduced Exercise Capacity

**Figure 1:**
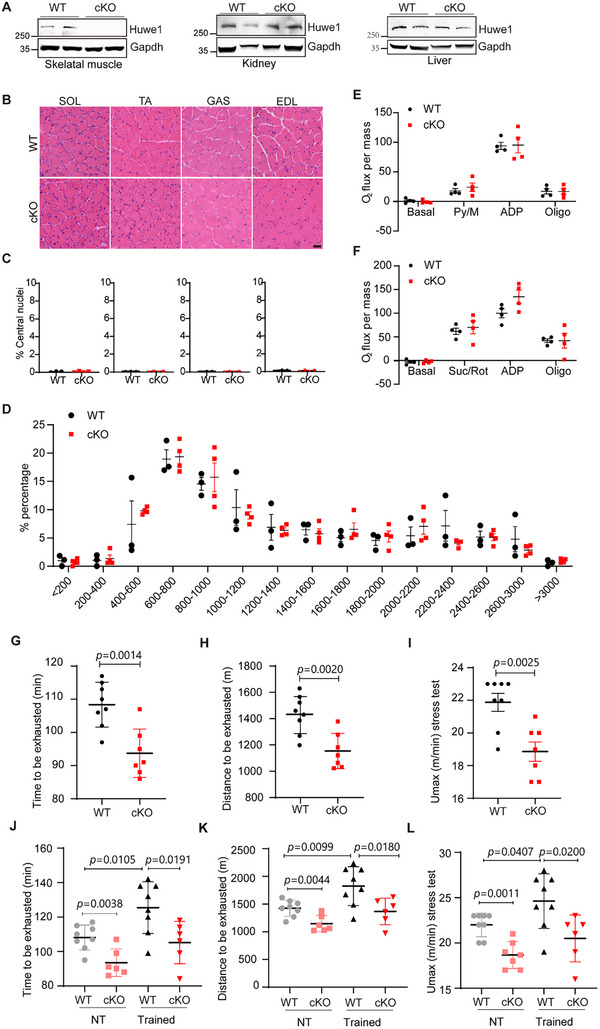
Mice with loss‐of‐function of Huwe1 in skeletal muscle display reduced exercise capacity. A) The expression of Huwe1 protein in different organs of WT and skeletal muscle‐specific *Huwe1* KO mice (cKO, n = 2). Gapdh served as the loading control; B,C) Hematoxylin and eosin (H&E) staining of soleus (SOL), tibialis anterior (TA), gastrocnemius (GAS), and extensor digitorum longus (EDL) from both WT and *Huwe1* cKO mice (B, scale bar, 50 µm). n = 4 in each group. Centrally located nuclei were quantified as means ± SEM (C); D) Quantified average fiber areas and percentages of fibers at different size intervals were shown as means ± SEM (n = 3–4 in each group), scale bar, 20 µm; E,F) Mitochondrial respiration rates were measured in soleus muscle of WT and *Huwe1* cKO mice using pyruvate (E) or succinate (F) as substrates (n = 4 for each group); G–L) WT and *Huwe1* cKO mice were subjected to one bout of exhausted exercise (n = 7–8, G‐I) or six‐week endurance training (n = 6 or 8, J–L). Running time (G, J), running distance (H, K), and maximum speed (I, L) of each mouse were recorded. Data are presented as Mean ± SEM. Student's t‐test was used to calculate the statistical probability (*p*) values shown between the indicated groups.

To further investigate the participation of HUWE1 in exercise, skeletal muscle‐specific Huwe1 knockout mice were developed by crossing *Huwe1* flox mice with myosin light polypeptide1 (*Myl1*)‐cre transgenic mice in which cre recombinase is selectively expressed in skeletal muscle (**Figure** **1**A). The resulting *Huwe1* ^fl/fl(y)^; *Myl1*‐Cre mice (referred to as cKO mice) exhibited remarkably little Huwe1 expression in the skeletal muscle without affecting its expression in other tissues, such as the kidney and liver (Figure 1A). On a regular chow diet, there was no remarkable difference on body weight or body composition between *Huwe1* cKO mice and their control littermates (Figure S2A,B, Supporting Information). Next, we checked whether loss of *Huwe1* influenced the morphology of skeletal muscle by microscopy. Hematoxylin and eosin (H&E) staining of skeletal muscles from the *Huwe1* cKO mice did not reveal any abnormalities as compared to the wildtype littermates. Rare existence of centrally located nuclei suggested no obvious damage and regeneration upon Huwe1 depletion (Figure 1B,C). Analysis of fiber size by immunofluorescence staining of dystrophin, which marks the perimeter of muscle fibers, revealed that Huwe1 cKO didn't influence the composition and distribution of fiber size from the small fibers to large fibers (Figure 1D; Figure S2C, Supporting Information).

Regarding to the energy supply, a metabolic cage was utilized to measure the oxygen consumption (VO_2_) and carbon dioxide production (VCO_2_). Twenty‐four‐hour oxygen consumption (VO_2max_) and Respiratory Exchange Ratio (RER) were similar between control and *Huwe1* cKO mice, in both the light and dark cycles (Figure S2D,E, Supporting Information). Moreover, transmission electron microscopy (TEM) was utilized to visualize the ultrastructure of the mitochondria, which revealed no obvious difference between WT and *Huwe1* cKO mice (Figure S2F, Supporting Information). Furthermore, mitochondrial respiration rates were determined in soleus muscles of *Huwe1* WT and cKO mice using pyruvate or succinate as substrates. Both pyruvate‐ and succinate‐driven mitochondrial respiration rates were similar in *Huwe1* cKO muscles compared to the WT controls (Figure 1E,F). We also analyzed the mRNA levels of key proteins in mitochondrial respiratory chain complexes in *Huwe1* cKO muscles, which showed no difference between *Huwe1* cKO mice compared to WT controls (Figure S2G, Supporting Information). These data suggested that no obvious abnormality was observed in mice with Huwe1 deficiency in skeletal muscle on a chow diet.

To explore the impact of *Huwe1* deletion on exercise capacity, we performed an exercise stress test in *Huwe1* cKO and control littermates, where mice ran at incrementally increasing speeds on a treadmill until exhaustion. Running time, running distance, and maximum speed of each mouse that can afford were recorded. Exercise capacity of *Huwe1* cKO mice was significantly decreased compared to control mice (Figure 1G–I; Figure S3A–C, Supporting Information). Moreover, *Huwe1* cKO mice behaved weaker than wild‐type mice in latency to fall in the rotarod test (Figure S3D, Supporting Information), while grip strength wasn't affected (Figure S3E, Supporting Information). Since endurance training was reported to improve exercise capacity, we asked if Huwe1 knockout would affect the exercise performance of mice post‐endurance training. As expected, after six‐week endurance training, the exercise capacity of control mice was obviously ameliorated (Figure 1J–L). However, *Huwe1* cKO mice only exhibited slight improvement; the difference on running performance between control and *Huwe1* cKO mice was even more robust (Figure 1J–L). Different muscle fiber types were reported to contribute to exercise capacity differently. However, it was not the case in our study, since the skeletal muscle displayed a normal and comparable composition of Type I and Type II fibers (Figure S3F, Supporting Information). And there were no differences in muscle‐fiber‐type specifically expressed genes (Type I: Myosin heavy chain 7 (Myh7) Gene Ontology; Type II: Myh1 and Myh4, Figure S3G, Supporting Information). Therefore, we concluded that mice with loss‐of‐function of Huwe1 in skeletal muscle display reduced exercise capacity.

### 2.3 Sln is Upregulated and Fpn is Downregulated in skeletal Muscle of Huwe1 Deficient mice, Leading to Blunted SERCA Activity and Accumulated Iron Content

**Figure 2:**
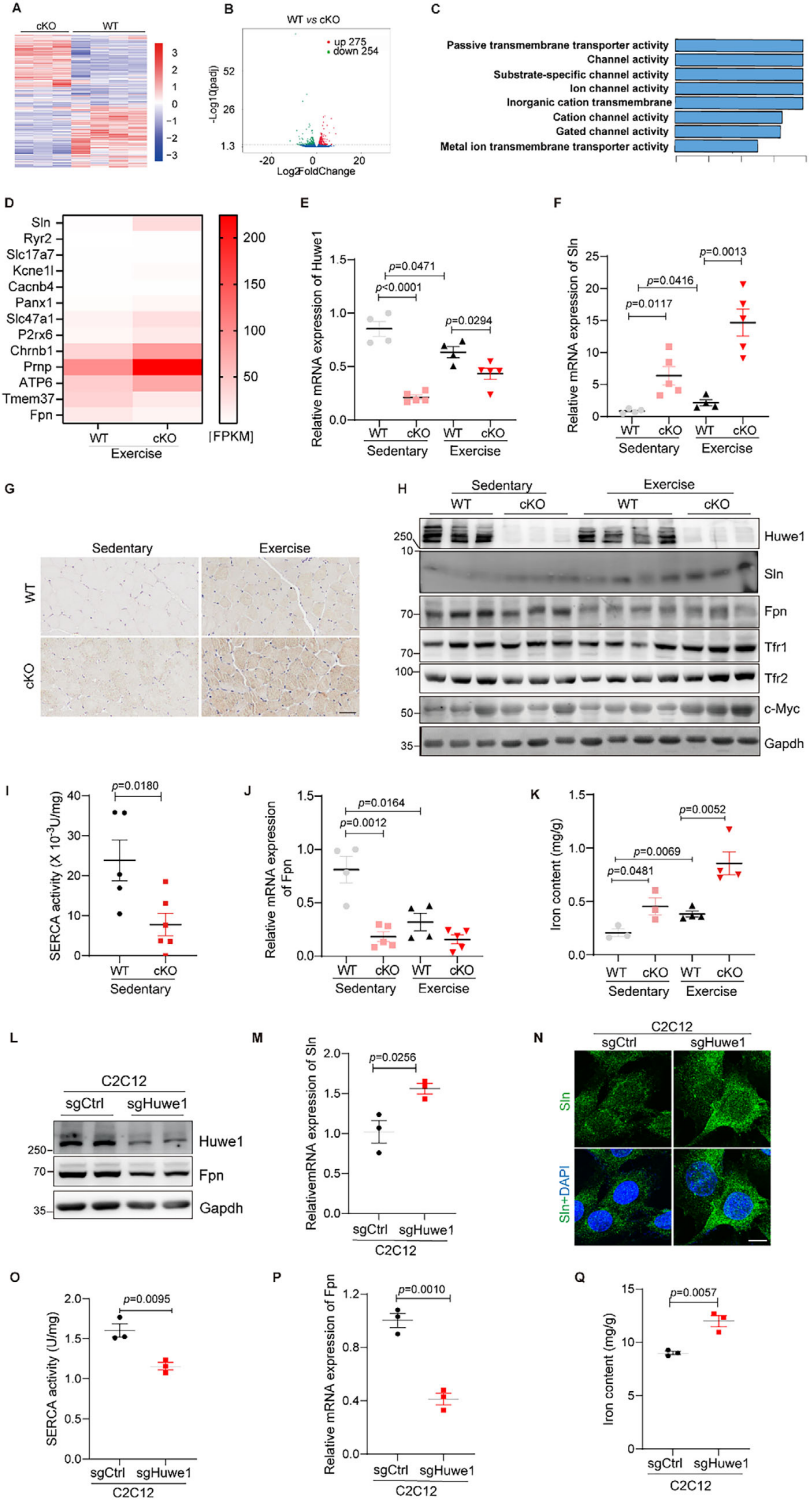
Sln is upregulated and Fpn is downregulated in skeletal muscle of Huwe1 deficient mice, leading to blunted SERCA activity and accumulated iron contents. A–C) RNA‐sequencing of soleus muscles of WT and *Huwe1* cKO mice after one bout of exhausted exercise (n = 3–4). Heat map (A), volcano plot (B), and GO enrichment (C) of differentially expressed genes (Down: log FC<−0.58 and *p* < 0.05, green; Up: log FC > 0.58 and *p* < 0.05, red); D) Heatmap of differentially expressed genes selected with ranking based on the fold changes; E,F) *Huwe1* ablation enhanced Sln expression. WT and *Huwe1* cKO mice were subjected to exhausted exercise (n = 4–5). Soleus muscles were collected and processed to assess the mRNA expression of Huwe1 (E) and Sln (F) by qPCR; G) Immunohistochemistry staining against Sln in soleus muscles from WT and *Huwe1* cKO mice under sedentary and exhausted exercise; Hematoxylin staining indicated the nuclei, scale bar, 100 µm; H) Immunoblots of Huwe1, Sln, Fpn, Tfr1, Tfr2, and c‐Myc in soleus muscles of WT and *Huwe1* cKO mice under sedentary and exhausted exercise (n = 3–4 in each group). Gapdh served as the loading control; I) SERCA activity of the soleus muscle was detected in WT and *Huwe1* cKO mice at sedentary (n = 5–6); J,K) The mRNA expression of Fpn (J) and the iron content (K) in soleus muscles of WT and *Huwe1* cKO mice subjected to exhausted exercise (n = 4–5); L–N) Huwe1 knockout enhanced Sln expression in C2C12 myoblast cells. Huwe1 was knocked out by specific sgRNAs in C2C12 cells (L). Sln expression in WT and Huwe1 KO C2C12 cells was analyzed by qPCR (M) and immunostaining against Sln (N). DAPI staining indicated nuclei. Images were obtained by confocal microscopy (Scale bar, 10 µm); O) SERCA activity was measured in WT and Huwe1 KO C2C12 cells; P) The mRNA levels of Fpn were detected in WT and Huwe1 KO C2C12 cells by qPCR; Q) Detection of iron content in control or Huwe1 knock‐out C2C12 cells. n = 2–3 in each group. Data are presented as Mean ± SEM. Student's t‐test was used to calculate the statistical probability (*p*) values shown between the indicated groups.

To further investigate the underlying molecular mechanisms in skeletal muscle upon *Huwe1* knockout, the soleus muscles from *Huwe1* cKO and WT mice with/without exhausted exercise were harvested for RNA‐sequencing based global transcriptome analysis. As indicated in the heatmap and volcano plot, a total of 529 genes were significantly differentially expressed after exhausted exercise, including 275 genes up‐regulated and 254 genes down‐regulated in the soleus muscles of *Huwe1* cKO mice compared to control group (Up: log FC > 0.58 and *p* < 0.05; Down: log FC <−0.58 and *p* < 0.05; **Figure** **2**A,B). Gene Ontology (GO) enrichment showed transmembrane transporter activity and the channel activity as the most abundant terms in the biological process categories (Figure 2C), in which Sln and Fpn/Slc40a1 represented the most significantly upregulated and downregulated genes, respectively (Figure 2D). Next, we detected the expression of Sln and Fpn in WT and *Huwe1* cKO mice upon exhausted exercise and endurance training to confirm these transcriptional changes. The mRNA level of Sln was more than ten‐old elevated in the skeletal muscle of *Huwe1* cKO mice subjected to both exhausted exercise and endurance training (Figure 2E,F; Figure S4A, Supporting Information). And the protein level of Sln was obviously higher in the muscle sections of *Huwe1* cKO mice subjected to both exhausted exercise and endurance training, although the ubiquitination of Sln in the soleus muscle was not affected by Huwe1 deletion (Figure 2G; Figure S4B,C, Supporting Information). It should be noted that other HUWE1 substrates, including  TfR1along with TfR2 in skeletal muscles, remained unchanged (Figure 2H). As reported, SLN is a small regulatory protein that inhibits SERCA pump.^[^ ^24^ ^]^ Overexpression of Sln decreases myocyte contractility and Ca^2+^ transport through inhibiting SERCA activity in skeletal muscle.^[^ ^25^ ^]^ Therefore, we evaluated the SERCA activity in the soleus and EDL muscles. As expected, SERCA activity of the soleus muscle but not the EDL was remarkably inhibited in *Huwe1* deficient mice, although SERCA2a expression was barely affected in the soleus muscle upon HUWE1 deletion (Figure 2I; Figure S4D–F, Supporting Information). Moreover, Sln was reported to regulate other calcium regulatory proteins, such as calcineurin.^[^ ^26^, ^27^ ^]^ We wondered if calcineurin signaling was affected by *Huwe1* deletion. In consistent with our observation that Sln was upregulated in the soleus muscle of *Huwe1* cKO mice, calcineurin signaling was also strengthened in the soleus but not the EDL muscles by Huwe1 deletion, suggesting that upregulated sarcolipin stimulated calcineurin signaling in the soleus muscles of *Huwe1* cKO mice (Figure S4G,H, Supporting Information).

On the other hand, both the mRNA and protein levels of Fpn were decreased in the soleus of *Huwe1* cKO mice compared with control littermates (Figure 2J; Figure S4G–I, Supporting Information). As an iron exporter, FPN plays a critical role in cellular iron release.^[^ ^28^ ^]^ FPN mutation (loss‐of‐function) reduced iron export from cells, which appears to be one of the common causes of hereditary iron overload.^[^ ^29^ ^]^ In consistence, *Huwe1* cKO mice with low expression of Fpn exhibited more iron accumulation (Figure 2K; Figure S4J,K, Supporting Information). The primary myoblasts were isolated from the soleus muscle of WT and *Huwe1* cKO mice, and then differentiated into myotubes (Figure S4L, Supporting Information). Consistent with in vivo data, c‐Myc was stabilized in myotubes from *Huwe1* cKO mice, with the upregulation of Sln and reduced SERCA activity (Figure S4M–O, Supporting Information). Moreover, Fpn was downregulated, accompanied with iron overload upon Huwe1 deletion (Figure S4P,Q, Supporting Information). Based on these observations, we tried to knock out *Huwe1* in C2C12 mouse myoblasts using Clustered regularly interspaced short palindromic repeats (CRISPR)/CRISPR‐associated protein 9 (Cas9) technology with specific guide RNA targeting mouse *Huwe1* (Figure 2L). Sln expression was increased in Huwe1 knock‐out C2C12 cells, accompanied with suppressed SERCA activity (Figure 2M–O). In the meantime, Fpn expression was inhibited and iron content was increased upon Huwe1 knock‐out in C2C12 cells (Figure 2P,Q).

In addition, 27 transcriptomic datasets of healthy humans subjected to disuse and exercise were analyzed to investigate the relationship between HUWE1 and SLN or FPN. Consistent with the observation that HUWE1 was downregulated upon exhausted exercise and endurance training, SLN was dramatically increased 1 hour post exhausted exercise, and FPN was highly inhibited vice versa (Figure S5A, Supporting Information). Moreover, the correlation between HUWE1 and SLN or FPN was also checked in the database of human skeletal muscle in GTEx, which demonstrated that HUWE1 is negatively correlated with SLN (R = −0.2, *p* = 7.6 ×0^−5^), and associated with FPN expression in a positive trend in skeletal muscle (Figure S5B, Supporting Information).

Taken together, these data demonstrated that Sln is upregulated and Fpn is decreased in soleus muscle of *Huwe1* deficient mice, leading to blunted SERCA activity and accumulated iron contents.

### 2.4 Silence of Huwe1 Modulates Sln and Fpn Expression via Stabilized c‐Myc Protein

**Figure 3:**
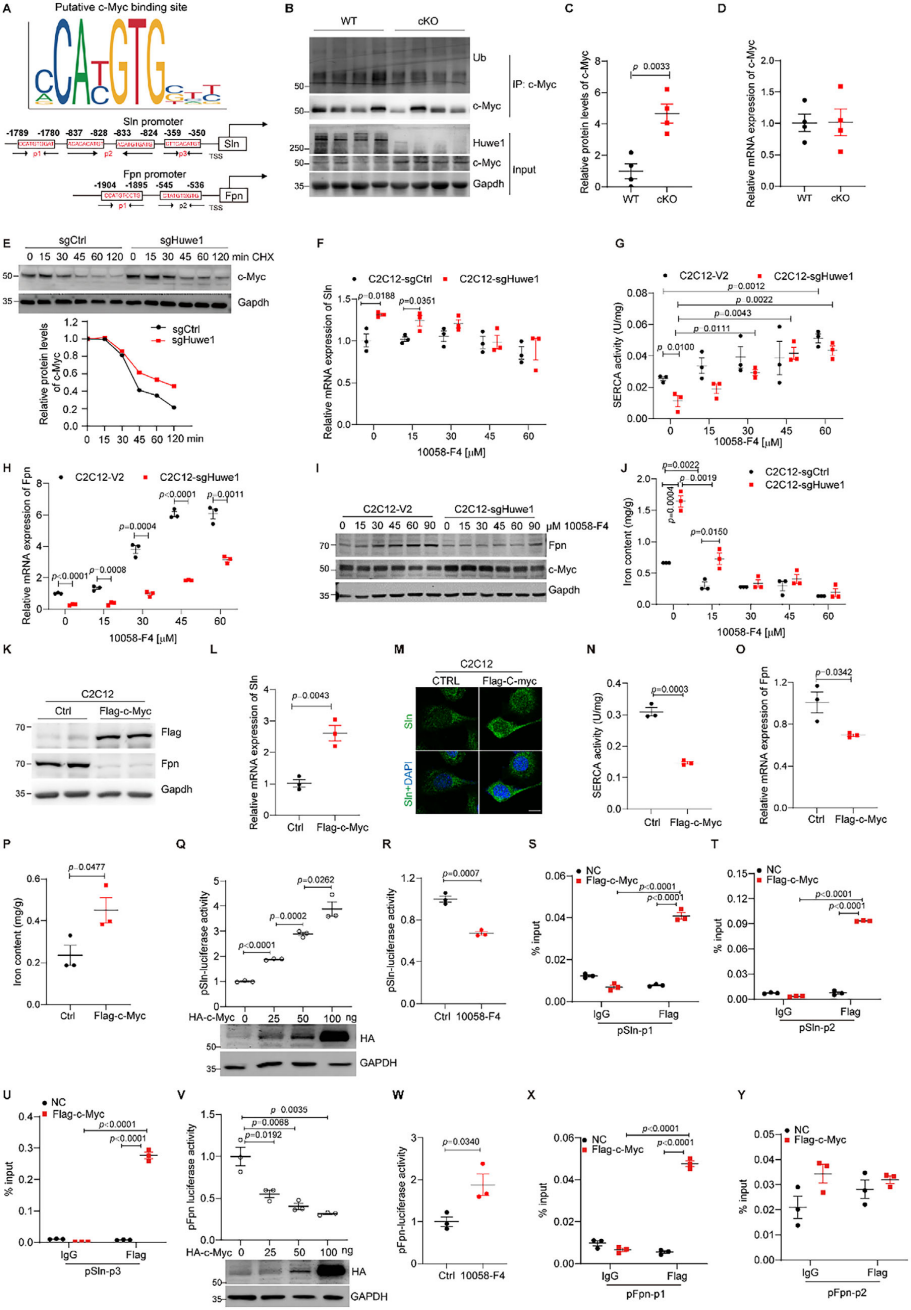
Silence of Huwe1 modulates Sln and Fpn expression via stabilized c‐Myc protein. A) Schematic representation to demonstrate the potential binding sites of c‐Myc in the promoter region of mouse *Sln* and *Fpn* genes. TSS, Transcription Start Site; p1, p2 or p3 indicates pairs of primers used for quantitative real‐time PCR in ChIP assay; B–D) c‐Myc is stabilized by *Huwe1* deletion in soleus muscles. c‐Myc was immunoprecipitated from soleus muscles of WT and *Huwe1* cKO mice, and its ubiquitination was detected by immunoblotting (n = 4 for each group, B). c‐Myc protein level in soleus muscle of WT and *Huwe1* cKO mice was quantified in the input samples of B (C). mRNA expression of c‐Myc was measured by qPCR in soleus muscles from WT and *Huwe1* cKO mice (n = 4, D); E) Immunoblots of c‐Myc in WT and Huwe1 KO C2C12 cells treated by cycloheximide (CHX, 100 µg ml^−1^) for the indicated times. c‐Myc and Gapdh blots were quantified by ImageJ, and Gapdh served as the loading control; F–J) WT and Huwe1 KO C2C12 cells were subjected to indicated concentrations of 10058‐F4 for 24 h. The expression of Sln was measured by qPCR (F), and SERCA activity was detected (G); The RNA and protein levels of Fpn were assessed by qPCR (H) and immunoblotting (I), and the iron content was determined (J); K–P) C2C12 cells were stably expressed with control (Ctrl) or Flag‐tagged c‐Myc (K). The expression of Sln was detected by qPCR (L) or immunofluorescence staining (M, scale bar, 10 µm); SERCA activity (N), mRNA expression of Fpn (O), and the iron content (P) were measured in control and c‐Myc overexpressed cells; Q,R) Sln promoter activity was measured in 293T cells transiently transfected with Sln promoter‐driven reporter plasmid and co‐transfected with increasing amount of HA‐tagged c‐Myc (Q) or treated with 10058‐F4 (60 µM for 24 h, R). Immunoblotting of HA indicates the amount of transfected c‐Myc (Q); S–U) ChIP‐qPCR assays were performed using antibodies against Flag or normal IgG with the indicated primers in Flag‐tagged c‐Myc over‐expressed C2C12 cells (Sln‐p1 in S, Sln‐p2 in T, and Sln‐p3 in U; corresponding occupied regions were labeled in A); V,W) Fpn promoter activity was measured in 293T cells transiently transfected with Fpn promoter‐driven reporter plasmid and co‐transfected with increasing amount of HA‐tagged c‐Myc (V) or treated with 10058‐F4 (60 µM for 24 h, W). Immunoblotting of HA indicates the amount of transfected c‐Myc (V); X,Y) Quantitative PCR tested the occupation of c‐Myc on Fpn promoter using antibodies against Flag or normal IgG with the indicated primers in Flag‐tagged c‐Myc over‐expressed C2C12 cells (Fpn‐p1 in X and Fpn‐p2 in Y; corresponding occupied region was labeled in A). Data are presented as Mean ± SEM (n = 3 in each group). Student's t‐test was used to calculate the statistical probability (*p*) values shown between the indicated groups.

To further understand the molecular mechanism of how Sln and Fpn were regulated, we attempted to identify the potential common transcription factors that could bind to the promoters of both *Sln* and *Fpn*, among which c‐Myc was previously reported as the substrate of ligase E3 HUWE1 (**Figure** **3**A).^[^ ^17^ ^]^ Meanwhile, a list of validated c‐Myc target genes was significantly dysregulated in soleus muscles of *Huwe1* cKO mice compared to wild‐type mice. Seven of these genes were inhibited and five of them were increased by Huwe1 deficiency, which was consistent with their transcriptional regulation by c‐Myc as previously reported.^[^ ^30^ ^]^ This observation suggested that the transcriptional activity of c‐Myc was increased in the soleus muscle of *Huwe1* cKO mice (Figure S6A, Supporting Information). Indeed, we observed that c‐Myc was accumulated at the protein level in the soleus muscle of *Huwe1* cKO mice while its mRNA expression remained almost unchanged (Figure 3B–D). Consistently, the half‐life of c‐Myc protein was prolonged in Huwe1 knockout cells compared to WT cells (Figure 3E). These data suggested that Huwe1 ubiquitinates c‐Myc in the soleus muscle for proteasome degradation.

Next, we tested the effect of c‐Myc on the expression of Sln and Fpn using pharmacological inhibitor or genetic ablation. As a small molecule inhibitor of Myc, 10058‐F4 dose‐dependently suppressed Sln expression with gradually enhanced SERCA activity, and abrogated the differences between WT and Huwe1 knock‐out C2C12 cells (Figure 3F,G). On the other hand, the decreased Fpn expression was dose‐dependently elevated in Huwe1 knock‐out cells treated by increased concentrations of 10058‐F4 (Figure 3H,I). Consistently, the high iron content in Huwe1 knock‐out cells was dramatically reduced to be comparable to that in control cells (Figure 3J). Meanwhile, we tried to suppress c‐Myc with specific small interfering RNAs (siRNAs) in C2C12 cells (Figure S6B, Supporting Information). The upregulation of Sln, as well as suppressed SERCA activity in Huwe1 knock‐out cells, was rescued by efficient silencing of c‐Myc (Figure S6C–E, Supporting Information). On the other hand, downregulated Fpn and the high iron content in Huwe1 deficient cells were also reversed upon c‐Myc silencing (Figure S6F,G, Supporting Information). These observations promoted us to further overexpress c‐Myc in C2C12 cells (Figure 3K). As expected, c‐Myc overexpression induced Sln expression with less SERCA activity (Figure 3L–N), and inhibited Fpn expression with more iron content (Figure 3O,P).

As we predicted the potential binding sites of c‐Myc on Sln promoter within 2001 base pairs upstream from their transcription start site (TSS), luciferase reporter constructs with the sequence from TSS to −2001 bp (pSln‐2001) contain its four putative binding sites. Sln promoter activity was dose‐dependently enhanced by c‐myc expression (Figure 3Q), and was significantly reduced by the small molecule inhibitor of Myc, 10058‐F4 (Figure 3R). Next, luciferase reporter constructs with the sequence from Sln TSS to −301 bp (pSln‐301), which doesn't contain the putative binding sites, were also tested along with pSln‐2001. As shown in Figure S6H,I (Supporting Information), cells transiently transfected with pSln‐2001 reporter showed a dose‐dependent increment in luciferase activity with c‐Myc over‐expression, and a remarkable decrease in luciferase activity treated by c‐Myc inhibitor 10058‐F4; However, cells transient transfected with pSln‐301 had no response to neither c‐Myc over‐expression nor 10058‐F4, which suggested the potential transcriptional regulation of Sln by c‐Myc. To get the direct evidence and further determine the specific binding sites of c‐Myc on the Sln promoter, we performed a chromatin immunoprecipitation (ChIP) assay in C2C12 cells with control or Flag‐tagged c‐Myc overexpression using antibodies against Flag. As shown in Figure 3S–U and c‐Myc exhibited a strong binding to −350 to −359 (Sln‐p3, primers 3) and relatively weak bindings to −1780 to −1789 (Sln‐p1, primers 1) and −824 to −837 bp (Sln‐p2, primers 2) upstream from its TSS. On the other hand, Fpn promoter activity was prominently decreased when c‐Myc was expressed (Figure 3V), but it was significantly enhanced following 10058‐F4 treatments (Figure 3W). Luciferase reporter constructs with the sequence from Fpn TSS to −2001 bp (pFpn‐2001) contain two predicted binding sites, while the one with the sequence from TSS to −501 bp (pFpn‐501) doesn't. Cells transiently transfected with pFpn‐2001 showed a suppressed luciferase activity in a dose‐dependent manner with c‐Myc over‐expression, while cells transient transfected with pFpn‐501 didn't respond to c‐Myc over‐expression (Figure S6J, Supporting Information). Furthermore, the ChIP assay showed that c‐Myc bound to the Fpn promoter region at −1895 to −1904 bp, rather than −536 to −545 bp upstream from its TSS (Figure 3X,Y). Taken together, these data suggested suppression of Huwe1 modulates Sln and Fpn expression via stabilized c‐Myc protein.

### 2.5 Oxidative Stress was Profoundly Induced Upon Ablation of Huwe1

**Figure 4:**
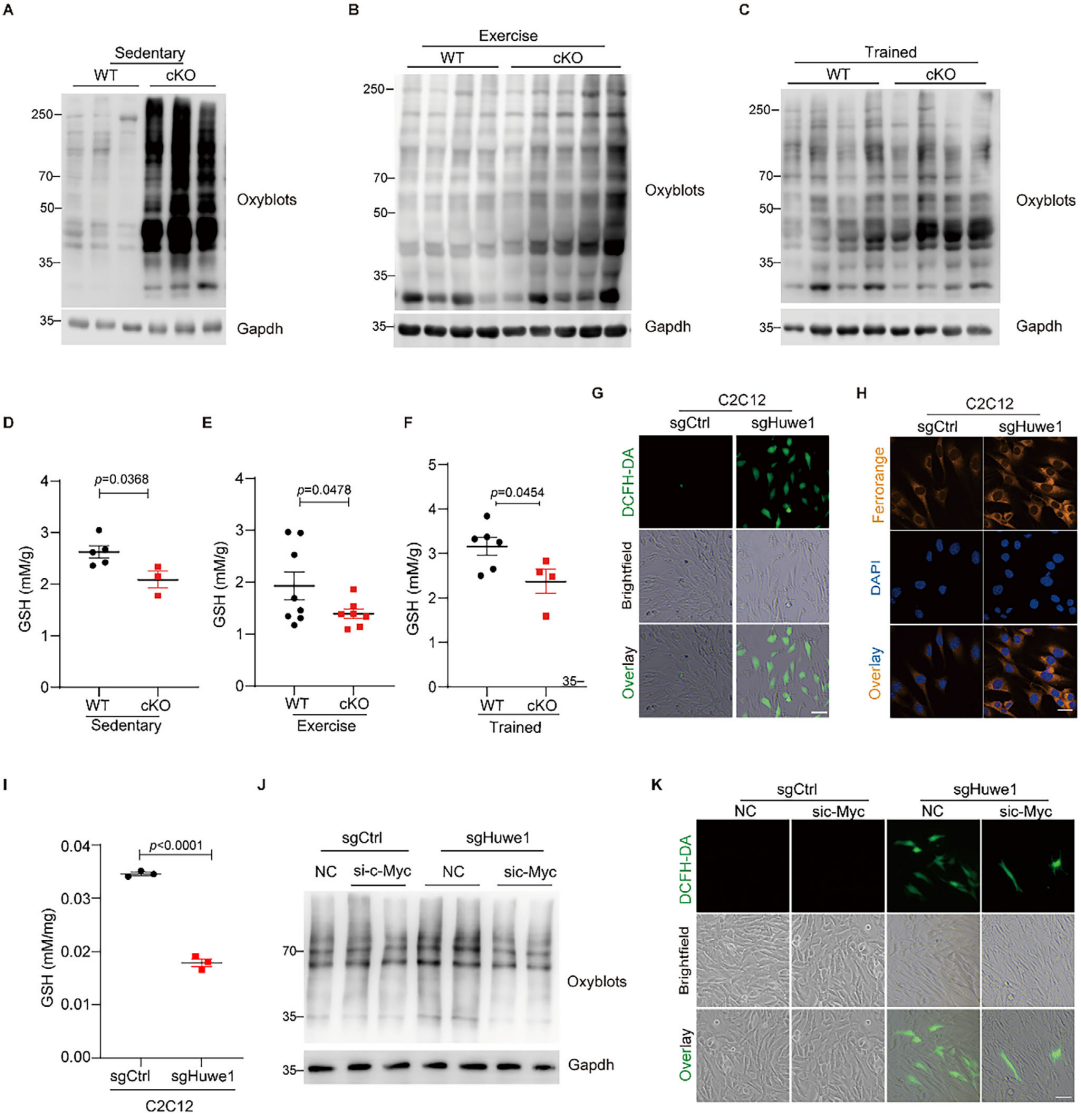
Oxidative stress was profoundly induced upon ablation of Huwe1. A–C) Protein carboxylation was elevated by Huwe1 deficiency. Oxyblots of soleus muscles in WT and *Huwe1* cKO mice under sedentary (A), exhausted exercise (B) and post endurance training (C), Gapdh served as an internal control (n = 3–5); D–F) GSH content was measured in soleus muscles of WT and *Huwe1* cKO mice under sedentary (D), exhausted exercise (E) and post endurance training (F) (n = 3–8); G,H) Representative fluorescent images of DCFH‐DA staining for general ROS (G), and confocal images of FerroOrange staining for the labile iron (H) in WT and Huwe1 KO C2C12 cells. Brightfield indicated the total cells to be stained, and DAPI staining indicated the nuclei. Scale bar, 25 µm; I) GSH content in WT and Huwe1 KO C2C12 cells (n = 3); J,K) WT and Huwe1 KO C2C12 cells were transfected with siRNA against c‐Myc for 48 h, then processed to measure protein carboxylation by oxyblots (J) and general cellular ROS by DCFH‐DA staining, brightfield indicated the total cells to be stained (K, scale bar, 25 µm). Data are presented as Mean ± SEM. Student's t‐test was used to calculate the statistical probability (*p*) values shown between the indicated groups.

Exercise‐induced muscle fatigue can occur under submaximal and maximal exercise intensity. Excessive free radicals, including reactive oxygen/nitrogen species (ROS/RNS) produced by exercise, can cause muscle oxidative stress and fatigue, which impacts exercise capacity and damages the body's health.^[^ ^31^, ^32^ ^]^ This, in turn, was associated with increased protein carbonylation‐a marker of protein oxidation and damage.^[^ ^33^ ^]^ Interestingly, the deletion of Huwe1 was accompanied by a remarkable induction of protein carbonylation even at sedentary conditions (**Figure** **4**A), which was consistent with the status of protein carbonylation after exhausted exercise and long‐term endurance training (Figure 4B,C). Glutathione in its reduced form (GSH)‐the most abundant non‐enzymatic antioxidant in the intracellular environment, acts as a primary indicator of the redox state and oxidative stress. Therefore, we assessed its redox state in *Huwe1* cKO mice and their control littermates before and after exercise. In line with the elevated protein carbonylation in *Huwe1* cKO skeletal muscle, GSH contents were significantly lower in *Huwe1* cKO mice compared to their control littermates (Figure 4D–F). These in vivo observations prompted us to confirm it in C2C12 myoblasts. General cellular ROS, as indicated by 2′,7′‐Dichlorofluorescein diacetate (DCFH‐DA) staining, was enhanced by *Huwe1* inhibition, accompanied with an increased labile Fe^2+^ pool (Figure 4G,H). GSH content was also decreased when Huwe1 was suppressed (Figure 4I).

Since silencing of Huwe1 modulates Sln and Fpn expressions via stabilized c‐Myc protein, we wondered whether c‐Myc was involved in the induction of protein oxidations and cellular ROS by the loss of Huwe1. As shown in Figure 4J, silence of c‐Myc abrogated the enhanced protein oxidation in Huwe1 knockout cells. Moreover, the robust cellular ROS in Huwe1 deficient cells was significantly inhibited by c‐Myc RNA interference (Figure 4K). These results implicated that ablation of Huwe1 in skeletal muscles confers a high oxidative status in which c‐Myc is a potent mediator.

### 2.6 Pharmacological Activation of SERCA or Silencing of Sln Ameliorates Exercise Performance in Huwe1 Deficient Mice

**Figure 5:**
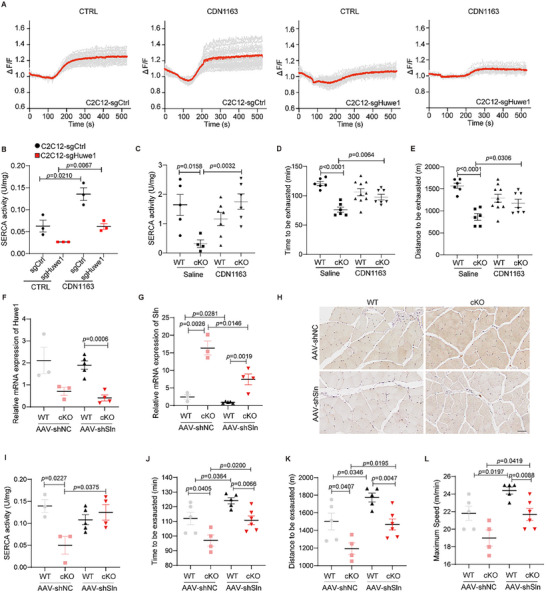
Pharmacological activation of SERCA or silencing of Sln ameliorates exercise performance in Huwe1 deficient mice. A,B) Time courses of Fura‐2 Ca^2+^ signals (F340/F380) in WT and Huwe1 KO C2C12 cells treated by CDN1163. WT and Huwe1 KO C2C12 cells were incubated with/without CDN1163 (10 µM) for 24 h. Afterward s, cells were subjected to recording the baseline of cytoplasmic Ca^2+^ by the Fura‐2 imaging approach under the microscope. And then ionomycin was added to monitor the total Ca^2+^ storage content. Individual cell trace (gray) and average trace (red) were shown for each group (A); SERCA activity was evaluated (B, n = 3); C–E) WT and *Huwe1* cKO mice were intraperitoneally injected with saline or CDN1163 (40 mg kg^−1^) for 7 consecutive days, followed by one bout of exhausted exercise; SERCA activity (C), running time (D) and running distance (E) of each mouse were recorded. n = 5–6 in each group; F–L) Adeno‐associated virus 2/9 (AAV2/9) packaged‐sh*Sln* and AAV2/9‐EGFP (shNC) were injected into the soleus and gastrocnemius muscles of each leg in WT and *Huwe1* cKO mice to knock down Sln, these mice were subjected to one bout of exhausted exercise 4 weeks after injection (n = 3–5 in each group). The expression of Huwe1 (F) and Sln (G) were detected by qPCR; Immunochemistry staining of Sln in indicated groups were shown and hematoxylin staining indicated the nuclei (H, scale bar, 100 µm); SERCA activities were assayed (I); Running time (J), running distance (K) and maximum speed (L) of each mouse in the indicated groups were recorded. Data are presented as Mean ± SEM. Student's t‐test was used to calculate the statistical probability (*p*) values shown between the indicated groups.

SERCA controls muscle relaxation by pumping Ca^2+^ from the cytosol into the lumen of SR, the main storage compartment for intracellular calcium.^[^ ^34^ ^]^ Given the accumulating evidence for the disturbance in cellular Ca^2+^ signaling caused by SERCA inactivation, we determined whether Ca^2+^ flux was altered in Huwe1 deficient cells. The Fura‐2 calcium imaging approach was used to estimate the total Ca^2+^ content by using ionomycin (IO) to elicit Ca^2+^ efflux from SR to the cytoplasm,^[^ ^35^ ^]^ and we found that Ca^2+^ storage in SR was dramatically decreased in Huwe1 KO C2C12 cells (**Figure** **5**A). Next, we investigated whether the upregulated Sln and inhibited SERCA activity contribute to the impaired exercise performance in Huwe1 deficient mice. CDN1163, an allosteric pan‐SERCA activator in vitro and in vivo,^[^ ^36^ ^]^ has been demonstrated to be potentially therapeutic in various animal models of oxidative stress‐related diseases, such as 6‐Hydroxydopamine hydrochloride (6‐OHDA)‐lesioned rats as a model of Parkinson's disease and superoxide dismutase 1 (SOD1)‐deficient mice.^[^ ^15^, ^37^ ^]^ Both the reduced SR calcium storage and inhibited SERCA activity in Huwe1 knockout cells were improved by CDN1163 treatment (Figure 5A,B). Importantly, the administration of CDN1163 (40 mg kg^−1^) significantly potentiated SERCA activity, especially in *Huwe1* cKO mice (Figure 5C). Accordingly, the exercise capacity was dramatically enhanced in *Huwe1* cKO mice upon CDN1163 treatment, as revealed by extended running time and running distance (Figure 5D,E).

Furthermore, we injected WT and *Huwe1* cKO mice with adeno‐associated virus (AAV) packaged with shRNA targeting mouse *Sln* to boost SERCA activity (Figure 5F–I). In consistency with the pharmacological activator, exercise performances were obviously improved upon Sln silencing, accompanied with ameliorated SERCA activity, especially in *Huwe1* cKO mice (Figure 5J–L). These results indicate that upregulated Sln with decreased SERCA activity is responsible for the impaired exercise performance in *Huwe1* cKO mice.

### 2.7 Dietary Iron Restriction Eliminates Oxidative Stress and Improves Exercise Performance in Huwe1 Deficient Mice

Our previous studies demonstrated that Huwe1 ablation impedes TfR11 degradation, which leads to aberrant iron accumulation and lipid peroxidation in liver injury.^[^ ^22^ ^]^ In line with the downregulation of Huwe1 upon exhausted exercise, iron content was indeed increased after exhausted exercise (Figure S7A, Supporting Information). Therefore, we continued to pursue the influence of accumulated iron on oxidative stress in the skeletal muscle of *Huwe1* cKO mice. Mice were fed with either a control diet (37.5 mg carbonyl Fe/kg, NID) or a high iron diet (8.3 g carbonyl Fe/kg; HID) for 8 weeks. To our surprise, the exercise capacity of *Huwe1* cKO mice was almost lost when fed with HID, while it's barely affected in WT mice (Figure S7B–D, Supporting Information). Meanwhile, more lipid peroxidation and protein oxidation were detected by malondialdehyde (MDA) content and protein carbonylation in the skeletal muscle of *Huwe1* cKO mice (Figure S7E,F, Supporting Information), suggesting that overwhelming iron might be the reason for the exercise weakness of *Huwe1* cKO mice.

**Figure 6:**
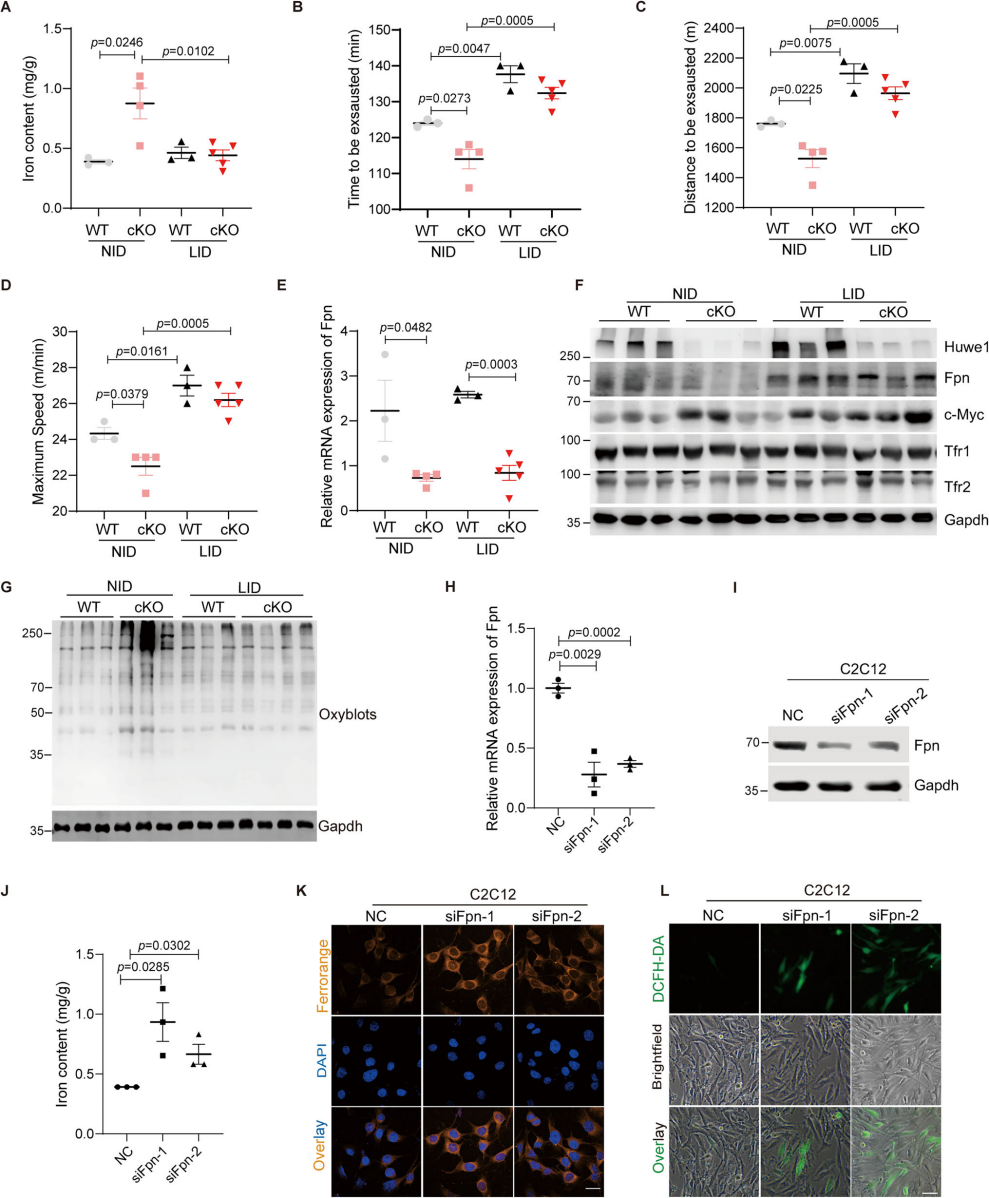
Dietary iron restriction eliminates oxidative stress and improves exercise performance in Huwe1 deficient mice. A–G) WT and *Huwe1* cKO mice were fed with a normal iron diet (NID, 37 mg carbonyl Fe/kg) or low iron diet (LID, 18.5 mg carbonyl Fe/kg) for 2 weeks, then subjected to one bout of exhausted exercise (n = 3–5 in each group). The iron content was measured in the soleus muscles of WT and *Huwe1* cKO mice (A); Running time (B), running distance (C) and maximum speed (D) of each mouse were recorded; The expression of Fpn was detected by qPCR (E); Immunoblots of Huwe1, Fpn, c‐Myc, Tfr1 and Tfr2 in soleus muscles of WT and *Huwe1* cKO mice fed with control diet (NID) or low iron diet (LID), Gapdh served as an internal control (F); Oxyblots of soleus muscles in WT and *Huwe1* cKO mice fed with NID or LID to measure protein carboxylation (G); H–L) C2C12 cells were transfected with negative control (NC) or two specific siRNAs targeting mouse *Fpn* for 48 h, then subjected to qPCR (H) and immunoblotting (I) of Fpn to confirm the knock‐down efficiency, n = 3 in each group; (J,K) Iron content (J, n = 3 in each group) and FerroOrange staining for the labile iron (K, scale bar, 25 µm) were operated to assay the impact of Fpn on iron accumulation; (L) General cellular ROS were detected by DCFH‐DA staining to evaluate the effect of Fpn on oxidative stress. Scale bar, 25 µm. Data are presented as Mean ± SEM. Student's t‐test was used to calculate the statistical probability (*p*) values shown between the indicated groups.

In parallel, mice were fed with either a control diet (37.5 mg carbonyl Fe/kg, NID) or a low iron diet (18.5 mg carbonyl Fe/kg, LID) for 2 weeks, which reduced the iron content in skeletal muscle of *Huwe1* cKO mice to be comparable to that in WT mice (**Figure** **6**A). Interestingly, exercise capacities in both groups with a low iron diet were remarkably ameliorated and even tended to be comparable (Figure 6B–D). It should be noted that although the mRNA level of Fpn remained lower in skeletal muscles of *Huwe1* cKO mice (Figure 6E), Fpn protein was stabilized by a low iron diet (Figure S7F, Supporting Information), which probably can explain the improvement of the iron overload in *Huwe1* cKO mice. In accordance, pronounced protein oxidation status in *Huwe1* cKO mice was abrogated by a low iron diet (Figure 6G), suggesting that elevated iron content in skeletal muscle of *Huwe1* cKO mice may play a vital role on oxidative stress. To further prove the effect of down‐regulated Fpn on iron overload and oxidative stress in Huwe1 deficient cells, we silenced Fpn by two different siRNAs in C2C12 cells (Figure 6H,I). Iron content was increased upon Fpn ablation (Figure 6J). Meanwhile, Fpn knockdown triggered more FerroOrange staining, an indicator of labile iron, which phenocopied the effect of Huwe1 knockout (Figure 6K). Moreover, increased cellular ROS induced by Huwe1 knockout was also mimicked by Fpn inhibition (Figure 6L). These findings suggested suppressed Fpn expression contributes to oxidative stress and iron overload in Huwe1 deficient mice.

In summary, our findings highlighted a crucial role for HUWE1 in the control of cellular iron and calcium homeostasis in skeletal muscle. We uncovered a novel mechanism involving in SLN and FPN expression via c‐MYC, through which HUWE1 coordinately regulates SERCA activity and iron homeostasis in exercise. This work provides novel insights into muscle weakness and could form the basis for the prevention and treatment of muscle fatigue.

## 3 Discussion

In our study, we observed downregulation of HUWE1 by acute exercise from published transcriptomic datasets and further confirmed this trend in mice, both of which prompted us to investigate whether HUWE1 could act as a regulator of exercise. To this end, we generated mouse lines in which the loss‐of‐function of Huwe1 was induced in a skeletal muscle‐specific manner (*Huwe1* cKO). *Huwe1* cKO mice displayed reduced exercise capacity with massive oxidative stress. Further mechanistic studies demonstrated that stabilized c‐Myc protein by Huwe1 deficiency upregulated Sln with decreased SERCA activity, and downregulated Fpn with increased iron accumulation. Thus, pharmacological activation of SERCA and silencing of Sln improved the exercise capacity of *Huwe1* cKO mice, and dietary iron restriction further mitigated oxidative stress in *Huwe1* cKO mice.

Exercise has been shown to profoundly benefit the quality of life and prevent chronic diseases for millions of people worldwide.^[^ ^38^, ^39^ ^]^ Exhausted exercise can induce excessive ROS production, leading to oxidative stress and muscle fatigue.^[^ ^40^ ^]^ Therefore, how the redox balance is delicately maintained during exercise is a crucial issue to be investigated. Our study links Huwe1 deficiency in skeletal muscle to the impairment of exercise capacity characterized by an enhanced accumulation of oxidative stress. Regarding to its function in oxidative stress, this is in line with our previous work, which reported HUWE1 as a protective factor against oxidative stress during acute liver injury.^[^ ^22^ ^]^ Of note, Huwe1 deficiency in skeletal muscle didn't influence mitochondrial energy metabolism as previously reported in the cardiomyocytes.^[^ ^41^ ^]^ Instead, transcriptomic analysis revealed transmembrane transporter activity as the most significantly dysregulated process by Huwe1 deficiency in skeletal muscle after exercise.

The calcium sequestering activity of SERCA, which facilitates muscular relaxation in both cardiac and skeletal muscle, is regulated at the level of protein content and is further modified by the endogenous proteins phospholamban (PLN) and SLN. It was reported that SERCA function was impaired in Sod1^−/−^ and Sod1^+/−^ mouse model of oxidative stress, and the allosteric SERCA activator CDN1163 ameliorated the muscle impairment.^[^ ^15^, ^42^ ^]^ In our study, we discovered the transcriptional upregulation of Sln and inhibition of SERCA activity by Huwe1 ablation. Interestingly, four putative c‐Myc binding sites were identified in the promoter of Sln, which was further approved that the Sln promoter‐driven luciferase activity was upregulated by c‐Myc overexpression. Moreover, both the chemical inhibition of c‐Myc by 10058‐F4 and the genetic inhibition by siRNAs specifically against c‐Myc remarkably reduced the upregulated Sln in Huwe1 deficient cells. These data unveiled the influence of HUWE1 in controlling Ca^2+^ homeostasis in skeletal muscle to coordinate exercise performance.

Skeletal muscle contains ≈10–15% of the iron in the body, which is essential for multiple processes that influence skeletal muscle performance, such as oxygen and electron transport.^[^ ^43^, ^44^ ^]^ Despite being the biggest tissue in the human body, the involvement of skeletal muscle in systemic iron homeostasis has been generally neglected. Cancer patients have been characterized by decreased iron availability in mitochondria, and iron supplementation was sufficient to preserve muscle function.^[^ ^45^ ^]^ As reported, muscles from old mice had increased iron levels, which were associated with increased susceptibility to ischemia‐reperfusion injury and impaired muscle regeneration.^[^ ^46^ ^]^ Herein, our data demonstrated that Huwe1 deficiency in the skeletal muscle leads to iron overload through downregulating Fpn, which exports iron from cells to plasma. Interestingly, dietary iron restriction reversed the reduced Fpn expression in *Huwe1* cKO mice, thereby alleviating the accumulated iron content and improving the exercise performance of *Huwe1* cKO mice. Considering that the mRNA expression of Fpn in *Huwe1* cKO mice remained remarkably lower when fed with a low iron diet (LID) compared to the WT group, it's reasonable to assume that the recovered Fpn protein expression in *Huwe1* cKO mice by LID occurred in a post‐transcriptional manner, which needs further investigation in the future.

Collectively, our findings demonstrate that skeletal muscle HUWE1 is a fundamental coordinator of exercise via regulating SLN/SERCA and FPN/Iron export axis. Given the important involvement of FPN in oxidative stress of skeletal muscle by our study, it could be a potential direction to be further investigated.

## 4 Experimental Section

### Mice

Male C57BL/6 mice (8–10 weeks old) were purchased from Shanghai SLAC Co., Ltd (Shanghai, China). *Huwe1* ^Flox/y^ mice were kindly provided by Dr. Zhenyue Hao and Dr. Tak Wah Mak (University Health Network, Toronto, Canada). *Huwe1* ^Flox^ mice were crossed with *Myl1*‐Cre (Jackson Laboratory, Bar Harbor, ME, USA) mice to generate *Huwe1* skeletal muscle‐specific knock‐out mice (*Huwe1* cKO). PCR‐based genotyping was performed using the primer pairs as previously described.^[^ ^47^ ^]^ All animals in the present study were bred in a standard environment with 12 h light/dark cycles and utilized for the experiments at the age of 8–10 weeks. All procedures related to animals were reviewed and approved by the Institutional Animal Care and Use Committee of Shanghai Jiao Tong University School of Medicine (APN: JUMC2023‐041‐A, Shanghai, China).

### Exhausted Exercise

Mice were exercised between the ages of 8–10 weeks on a treadmill (Anhui Zhenghua Biologic Apparatus Facilities Co., Ltd). On the first two days before exercise, mice were acclimated to the treadmill for 5 min at the speed of 8 or 10m min^−1^, respectively. Subsequently, mice were then assigned to a sedentary control (Sed) and an exhausted exercise group. Food was withdrawn overnight for both groups. On the third day, mice were subjected to a single bout of running, starting at the speed of 10 m min^−1^. Forty minutes later, the speed was increased at a rate of 1 m min^−1^ every 10 min for a total of 30 min. Then, the speed was increased at the rate of 1 m min^−1^ every 5 min until mice were exhausted, which was defined by inability to remain on the treadmill up to 5 s. Animals were removed immediately after exhaustion. Total running time, running distance, and maximum speed that they could afford were recorded for each mouse of wildtype and *Huwe1* cKO mice. The soleus muscle was used for the following measurements and staining.

### Endurance Training

Male C57BL/6 mice and mice from both genotypes (8–10 weeks old) were allocated into sedentary and training groups. The training group underwent all bouts of exercise 5 days per week and up to 6 weeks for endurance training, while the sedentary group remained sedentary on the treadmill for the same period of time. Animals ran 12 m min^−1^ for 30 min day^−1^ with 5° incline for the first week, followed by an increase to 40 min day^−1^ in the second and subsequent weeks. All bouts of exercise were performed at the start of the dark cycle to minimize disruption to the circadian rhythm.

### Animal Treatments

For CDN1163 injection, CDN1163 was purchased from TargetMol and was intraperitoneally injected into mice at 40 mg kg^−1^ body weight for 7 days before conducting the exhausted exercise. For high‐iron diet treatment, mice were fed with standard AIN‐76A diet containing 37 mg carbonyl iron/kg normal iron diet (NID, Dyets, Bethlehem, PA, USA) or the high‐iron diet (HID; 8.3 g carbonyl iron/kg) for 8 weeks. For low‐iron diet treatment, mice were fed with standard AIN‐76A diet containing 37 mg carbonyl iron/kg normal iron diet (NID, Dyets, Bethlehem, PA, USA) or the low‐iron diet (LID; 18.5 mg carbonyl iron/kg) for 2 weeks. Mice from both genotypes were assigned for exhausted exercise after the treatment.

### RNA‐Seq Studies

Transcriptomics analyses were performed using RNA‐sequencing as described previously.^[^ ^48^ ^]^ Total RNA was isolated from the entire soleus muscle of 8‐week‐old male *Huwe1* cKO and WT control mice using TRIzol (Invitrogen). RNA‐seq using Illumina HiSeq 4000 was performed by Beijing Novogene Bioinformatics Technology Co., Ltd. Three independent samples per group were analyzed. Paired‐end, 150 nt reads were obtained from the same sequencing lane. Transcriptome sequencing libraries averaged 39 million paired reads per sample, with 87.1% alignment to the mouse genome (UCSC mm10). The sequencing reads were then aligned to the UCSC mm10 genome assembly using TopHat 2.0.14 with the default parameters. Fragments Per Kb of exon per Million mapped reads (FPKM) were calculated using Cufflinks 2.2.1. The criteria for a regulated gene were a fold change greater than 1.5 (either direction) and a significant *p*‐value (<0.05) versus WT. For pathway analysis, the filtered data sets were uploaded into DAVID Bioinformatics Resources 6.8 to review the bio pathways using the Functional Categories database. The GO analysis was used to interpret data, and the regulated terms were ranked by *p*‐value. Heatmaps were generated using Morpheus (https://software.broadinstitute.org/morpheus).

### AAV‐shSln Production, Purification, and Injection

AAV2/9‐shSln‐EGFP was produced and purified by Hanbio Biotechnology (Shanghai, China) using sequence 5′‐CAGUGAGCCUUAGCUUUGUTT‐3′ specifically targeting to Sln. And AAV2/9‐shNC‐EGFP acted as a negative control by using sequence 5′‐UUCUCCGAACGUGUCACGUTT‐3′. To knock down Sln in vivo, mice were injected in the soleus and gastrocnemius muscles of both legs with a dose of 6.0 × 10^10^ genome copy (GC) of AAV2/9‐shSln. Mice injected with AAV2/9‐EGFP at a dose of 6.0 × 10^10^ GC performed as control groups. After 4 weeks of recovery, exhausted exercise was conducted.

### SERCA Activity, Iron Content, and GSH Content

Soleus muscles were freshly isolated and rapidly homogenized with saline in a homogenizer (Jingxing, Shanghai, China) under 4 °C. C2C12 cells were lysed in TAP buffer (20 mM Tris‐HCl with pH 7.5, 150 mM NaCl, 0.5% NP‐40, 1 mM NaF, 1 mM Na_3_VO_4_, 1 mM EDTA, and protease inhibitors). Supernatants were collected after centrifugation at 12 000 g for 10 min. SERCA activity and iron content were measured by using the Ca^2+^‐ATPase Assay Kit (Nanjing Jiancheng Bioengineering Institute, China) and the Tissue Iron Assay Kit (Nanjing Jiancheng Bioengineering Institute) according to the manufacturer's instructions. GSH content was measured by using the GSH/GSSG‐Glo Assay kit according to the manufacturer's instructions (Promega, USA).

### Cell Culture and Chemicals

The C2C12 myoblast cells were kindly provided by Dr. Liming Sun (Center for Excellence in Molecular Cell Science, Chinese Academy of Sciences, Shanghai, China). Human embryonic kidney HEK293T cells were purchased from the American Type Culture Collection (ATCC) and were authenticated by the manufacturer. No additional authentication was performed by the authors for any of the cell lines. These cells were maintained at 37 °C in a humidified incubator with 5% CO_2_ and cultured in Dulbecco's modified Eagle's medium (DMEM, Sigma) supplemented with 10% FBS (FSP500, ExCell Bio, Suzhou, China), 100 U mL^−1^ penicillin, and 10 µg mL^−1^ streptomycin (SV30010, HyClone Laboratories, USA). For pharmacologic c‐Myc inhibition, cells were incubated with 10058‐F4 (Beyotime) for 24 h at the indicated concentration. For protein stability evaluation, cells were treated with 100 µg ml^−1^ Cycloheximide (MedChemExpress). *Huwe1* knock‐out sublines were established by transducing parental C2C12 cells with lentivirus packaged with guild RNA targeting mouse *Huwe1*. c‐Myc overexpressed sublines were established by transducing parental C2C12 cells with lentivirus packaged with mouse c‐Myc. C2C12 were selected with puromycin at 2 µg ml^−1^. Only Mycoplasma noncontaminated cells were used for experiments. The cell lines were used between passages 2 and 15.

### sgRNA, Plasmids, and siRNA Transfection

*Huwe1* sgRNA (5′‐GGACCUGUUGGACCGCUUCG‐3′) was cloned into lentiCRISPR‐v2 (Addgene #52961). cDNAs of mouse *c‐Myc* were obtained from the Plasmid Library of Bio‐Research Innovation Center, Suzhou (China). *c‐Myc* was cloned into pCDH‐CMV‐MCS‐EF1‐Flag‐Puro (CD510B‐1) and pCDNA4.0‐HA. siRNAs targeting mouse *c‐Myc* were purchased from Dharmacon‐Horizon Discovery. Two siRNAs targeting mouse Fpn/Slc40a1 (5′‐GCCUGGCUUUCCUCUAUAUTT‐3′; 5′‐GCCCAGCUUUCCUGUUUAATT‐3′) and a non‐targeting siRNA (5′‐UUCUCCGAACGUGUCACGUTT‐3′) were synthesized by Shanghai GenePharma Co., Ltd (Shanghai, China). Transfections of siRNAs were performed using Lipofectamine RNAiMax (Invitrogen) according to the manufacturer's instructions.

### Western Blot

Proteins were extracted with RIPA buffer (50 mM Tris‐HCl with pH 7.4; 150 mM NaCl; 0.1% SDS; 2 mM EDTA; 50 mM NaF; 0.5% Deoxycholate and 1% NP‐40), containing Protease Inhibitor Cocktail (APExBIO, Houston, TX, USA). Protein concentration was quantified by using the BCA Protein Assay Kit (Thermo Fisher Scientific, Waltham, MA, USA). 30 µg of protein lysates were subjected to SDS‐PAGE. Immunoblot analysis was performed by using antibodies specific to HUWE1/Lasu1 (Bethyl Laboratories, Montgomery, USA), Fpn/Slc40a1 (Alpha Diagnostic International), c‐Myc (Santa Cruz Biotechnology), Tfr1 (Abclonal), Tfr2 (Signalway Antibody), Flag (Sigma‐Aldrich), and GAPDH (Proteintech). Protein oxidation in soleus skeletal muscle and C2C12 myoblasts was assessed by immunoblotting using the OxyBlot Protein Oxidation Detection Kit according to the manufacturer's instructions (Merck Millipore, Burlington, MA).

### Real‐Time Quantitative PCR

The total RNA was extracted from mice skeletal tissues or C2C12 cells using TRIzol (Invitrogen). cDNA was synthesized using the Superscript II reverse transcriptase Kit (Vazyme) via 1 µg of total RNA. The cDNAs were amplified using specific primers and BrightCycle Universal SYBR Green qPCR Mix with UDG (for C2C12 cells, Abclonal) or Genious 2X SYBR Green Fast qPCR Mix (for mice samples, Abclonal) at the thermal cycler Quant Studio 3 (Applied Biosystems). Gene expression was normalized to Rplp0. The primers were listed in the Supplementary Table S1 (Supporting Information).

### Mitochondrial Respiration Rates Measurement

Mitochondrial respiration rates were measured in soleus muscles with pyruvate or succinate as substrates as described previously.^[^ ^48^ ^]^ Briefly, the whole soleus muscle was dissected and permeabilized with 25 µg ml^−1^ saponin in BIOPS solution (7.23 mM K_2_EGTA, 2.77 mM CaK_2_EGTA, 20 mM imidazole, 20 mM taurine, 50 mM potassium 2‐(N‐morpholino)‐ethanesulfonic acid, 0.5 mM dithiothreitol, 6.56 mM MgCl_2_, 5.7 mM ATP, and 14.3 mM phosphocreatine (pH 7.1)). Measurement of oxygen consumption in permeabilized muscle fibers bundles were performed in buffer Z (105 mM potassium 2‐(N‐morpholino)‐ethanesulfonic acid, 30 mM KCl, 10 mM KH_2_PO_4_, 5 mM MgCl_2_, 5 mg ml^−1^ bovine serum albumin and 1 mM EGTA (pH 7.4)) at 37 °C and in the oxygen concentration range 400–250 nmol O_2_ per ml in the respiration chambers of an Oxygraph‐2k (Oroboros). Following measurement of basal, pyruvate (10 mM)/malate (5 mM), and succinate (5 mM)/rotenone (10 µM) respiration, maximal (ADP stimulated) respiration was determined by exposing the mitochondria to 4 mM ADP. Uncoupled respiration was evaluated following the addition of oligomycin (1 µg ml^−1^). Respiration rates were determined and normalized to tissue wet weight using DatLab 5 software (Oroboros Inc., Innsbruck, Austria), and the data were expressed as “pmol O_2_ s^−1^ mg wet weight^−1^.”

### C2C12 Immunofluorescence Staining

For immunofluorescence analysis, cells cultured on cover glasses were fixed with 4% paraformaldehyde for 10 min, followed by methanol at −20 °C for 10 min. Cells were incubated with Sln antibody (Proteintech) overnight at 4 °C, washed three times with PBS, and then incubated with goat anti‐rabbit Alexa Fluor 488 (Life Technologies) secondary antibody for 1 h at room temperature, followed by DAPI solution for 10 min to stain the nuclei. Cells were washed three times with PBS and mounted with Prolong Diamond (Invitrogen). Mitochondria were labeled with MitoTracker Orange CMTMRos (Invitrogen) according to the manufacturer's protocol. Labile ferrous ions in cells were also imaged by FerroOrange (Dojingo, Japan) according to the manufacturer's instructions. Cells were mounted with Prolong Diamond (Invitrogen). DNA was visualized by DAPI staining. Cells were imaged by an FV3000 Laser Scanning Confocal Microscope (Olympus, Tokyo, Japan). Total reactive oxygen species (ROS) was detected as described^[^ ^49^ ^]^ by using the ROS assay kit (Dojingo, Japan). Cells were incubated in DCFH‐DA working solution for 30 min at 37 °C. After wash in DMEM for 3 times, representative fluorescent images were taken using the FITC channel on a fluorescence microscope (Eclipse Ti2, Nikon, Japan).

### ER Ca^2+^ Efflux

A Fura‐2 calcium imaging approach to estimate the total Ca^2+^ store content by eliciting Ca^2+^ efflux from ER to the cytoplasm with ionomycin (IO) treatment was used, which can be used to indicate SERCA activity of ER. Briefly, C2C12 cells in 6‐well plates were incubated with Hank`s Balanced Salt Solution (HBSS, 14025–092, Gibco) with 1 µM Fura‐2 (Thermo Fisher) for 30 min. After loading, the cell glass coverslips were moved to a glass chamber and fixed. C2C12 cells were first immersed in calcium‐free HBSS (14170112, Gibco) for 100 s to record the baseline values, and then calcium‐free HBSS with 1 µM IO (Beyotime) was added. The ratios of Fura‐2 intensities at excitation wavelengths 340 and 380 nm (R340/380) were taken as measures of the Ca^2+^ pool.

### Statistical Analysis

All assays were performed at least 3 times, and reproducible results were obtained to make the conclusions. Statistical analysis was performed using a two‐tailed unpaired Student's t‐test as stated (Prism; GraphPad), and the statistical probability (*p*) value was shown in all the figures between the indicated groups. Results were presented as Mean ± SEM. No statistical methods were used to predetermine sample sizes, and sample size was explicitly stated in the figure legends. All data points were used in statistical analyses. Correlation of protein expressions in the database of human skeletal muscle in GTEx was statistically analyzed by the Pearson Correlation Coefficient method. ImageJ was used for blot quantification. For muscle sections, fiber size and numbers of satellite cells were recognized by the Myosight plugin in ImageJ^[^ ^50^ ^]^ which can be obtained from: https://imagej.net/Fiji/Downloads and https://github.com/LyleBabcock/MyoSight.

Additional experimental procedures are described in the **Supplementary Data**.

## Conflict of Interest

The authors declare no conflict of interest.

## Author Contributions

H.J. and Y.D. contributed equally to the work. Y.D. started the project and H.J. performed most of the experiments with the assistance from X.G. and J.L. D.Z. measured mitochondrial respiration rates with the guidance from Dr. Z.G. Z.H. provided the *Huwe1* ^flox^ mice. Dr. N.X. helped with the ER Ca^2+^ efflux imaging. H.J., Y.H., and J.Z. conceived the project, designed the experiments, analyzed the data and wrote the manuscript with the assistance of all authors. All authors discussed the results and commented on the manuscript.

## Supporting information



Supporting Information

## Data Availability

The data that support the findings of this study are openly available in NCBI's Sequence Read Archive (SRA SRP645028) database https://trace.ncbi.nlm.nih.gov/Traces/?view=study&acc=SRP645028.

## References

[1] F. W. Booth , M. V. Chakravarthy , S. E. Gordon , E. E. Spangenburg , J. Appl. Physiol. 2002, 93, 3. 12070181 10.1152/japplphysiol.00073.2002

[2] S. K. Powers , Z. Radak , L. L. Ji , J. Physiol. 2016, 594, 5081. 26893258 10.1113/JP270646 PMC5023699

[3] S. K. Powers , M. J. Jackson , Physiol. Rev. 2008, 88, 1243. 18923182 10.1152/physrev.00031.2007 PMC2909187

[4] S. K. Powers , L. L. Ji , A. N. Kavazis , M. J. Jackson , Compr. Physiol. 2011, 1, 941. 23737208 10.1002/cphy.c100054 PMC3893116

[5] S. Abboud , D. J. Haile , J. Biol. Chem. 2000, 275, 19906. 10747949 10.1074/jbc.M000713200

[6] A. Donovan , A. Brownlie , Y. Zhou , J. Shepard , S. J. Pratt , J. Moynihan , B. H. Paw , A. Drejer , B. Barut , A. Zapata , T. C. Law , C. Brugnara , S. E. Lux , G. S. Pinkus , J. L. Pinkus , P. D. Kingsley , J. Palis , M. D. Fleming , N. C. Andrews , L. I. Zon , Nature 2000, 403, 776. 10693807 10.1038/35001596

[7] A. T. McKie , P. Marciani , A. Rolfs , K. Brennan , K. Wehr , D. Barrow , S. Miret , A. Bomford , T. J. Peters , F. Farzaneh , M. A. Hediger , M. W. Hentze , R. J. Simpson , Mol. Cell 2000, 5, 299. 10882071 10.1016/s1097-2765(00)80425-6

[8] A. Donovan , C. A. Lima , J. L. Pinkus , G. S. Pinkus , L. I. Zon , S. Robine , N. C. Andrews , Cell Metab. 2005, 1, 191. 16054062 10.1016/j.cmet.2005.01.003

[9] A. Polonifi , M. Politou , V. Kalotychou , K. Xiromeritis , M. Tsironi , V. Berdoukas , G. Vaiopoulos , A. Aessopos , Blood Cells, Mol., Dis. 2010, 45, 233. 20691620 10.1016/j.bcmd.2010.07.002

[10] T. F. Reardon , D. G. Allen , Exp. Physiol. 2009, 94, 720. 19201785 10.1113/expphysiol.2008.046045

[11] S. Ali , J. M. S. Garcia , Gerontology 2014, 60, 294. 24731978 10.1159/000356760 PMC4112511

[12] S. Boncompagni , L. d'Amelio , S. Fulle , G. Fano , F. Protasi , J. Gerontol., Ser. A 2006, 61, 995. 10.1093/gerona/61.10.995 17077192

[13] M. Asahi , Y. Sugita , K. Kurzydlowski , S. De Leon , M. Tada , C. Toyoshima , D. H. MacLennan , Proc. Natl. Acad. Sci. U S A 2003, 100, 5040. 12692302 10.1073/pnas.0330962100 PMC154294

[14] H. Xu , H. Van Remmen , Skeletal Muscle 2021, 11, 25. 34772465 10.1186/s13395-021-00280-7 PMC8588740

[15] R. Qaisar , S. Bhaskaran , R. Ranjit , K. Sataranatarajan , P. Premkumar , K. Huseman , H. Van Remmen , Redox Biol. 2019, 20, 68. 30296699 10.1016/j.redox.2018.09.018 PMC6174848

[16] M. J. Friez , S. S. Brooks , R. E. Stevenson , M. Field , M. J. Basehore , L. C. Adès , C. Sebold , S. McGee , S. Saxon , C. Skinner , M. E. Craig , L. Murray , R. J. Simensen , Y. Y. Yap , M. A. Shaw , A. Gardner , M. Corbett , R. Kumar , M. Bosshard , B. van Loon , P. S. Tarpey , F. Abidi , J. Gecz , C. E. Schwartz , BMJ Open 2016, 6, 009537. 10.1136/bmjopen-2015-009537 PMC4854010 27130160

[17] S. Adhikary , F. Marinoni , A. Hock , E. Hulleman , N. Popov , R. Beier , S. Bernard , M. Quarto , M. Capra , S. Goettig , U. Kogel , M. Scheffner , K. Helin , M. Eilers , Cell 2005, 123, 409. 16269333 10.1016/j.cell.2005.08.016

[18] D. Chen , N. Kon , M. Li , W. Zhang , J. Qin , W. Gu , Cell 2005, 121, 1071. 15989956 10.1016/j.cell.2005.03.037

[19] Q. Zhong , W. Gao , F. Du , X. Wang , Cell 2005, 121, 1085. 15989957 10.1016/j.cell.2005.06.009

[20] J. Zhang , S. Kan , B. Huang , Z. Hao , T. W. Mak , Q. Zhong , Genes Dev. 2011, 25, 2610. 22016339 10.1101/gad.170605.111 PMC3248682

[21] S. H. Kao , H. T. Wu , K. J. Wu , J. Biomed. Sci. 2018, 25, 67. 30176860 10.1186/s12929-018-0470-0 PMC6122628

[22] Y. Wu , H. Jiao , Y. Yue , K. He , Y. Jin , J. Zhang , J. Zhang , Y. Wei , H. Luo , Z. Hao , X. Zhao , Q. Xia , Q. Zhong , J. Zhang , Cell Death Differ. 2022, 29, 1705. 35260822 10.1038/s41418-022-00957-6 PMC9433446

[23] P. A. Makhnovskii , R. O. Bokov , F. A. Kolpakov , D. V. Popov , Int. J. Mol. Sci. 2021, 22, 1208. 33530535 10.3390/ijms22031208 PMC7866200

[24] L. M. Espinoza‐Fonseca , J. M. Autry , D. D. Thomas , Biochem. Biophys. Res. Commun. 2015, 463, 37. 25983321 10.1016/j.bbrc.2015.05.012 PMC4465059

[25] G. Babu , Z. Zheng , P. Natarajan , D. Wheeler , P. Janssen , M. Periasamy , Cardiovasc. Res. 2005, 65, 177. 15621045 10.1016/j.cardiores.2004.08.012

[26] V. A. Fajardo , B. A. Rietze , P. J. Chambers , C. Bellissimo , E. Bombardier , J. Quadrilatero , A. R. Tupling , Am. J. Physiol. Cell Physiol. 2017, 313, C154. 28592414 10.1152/ajpcell.00291.2016

[27] V. A. Fajardo , P. J. Chambers , E. S. Juracic , B. A. Rietze , D. Gamu , C. Bellissimo , F. Kwon , J. Quadrilatero , A. Russell Tupling , Hum. Mol. Genet. 2018, 27, 4094. 30137316 10.1093/hmg/ddy302 PMC6240731

[28] A. Donovan , C. A. Lima , J. L. Pinkus , G. S. Pinkus , L. I. Zon , S. Robine , N. C. Andrews , Cell Metab. 2005, 1, 191. 16054062 10.1016/j.cmet.2005.01.003

[29] K. Pantopoulos , Front. Nutr. 2018, 5, 103. 30420953 10.3389/fnut.2018.00103 PMC6215844

[30] S. Walz , F. Lorenzin , J. Morton , K. E. Wiese , B. von Eyss , S. Herold , L. Rycak , H. Dumay‐Odelot , S. Karim , M. Bartkuhn , F. Roels , T. Wüstefeld , M. Fischer , M. Teichmann , L. Zender , C.‐L. Wei , O. Sansom , E. Wolf , M. Eilers , Nature 2014, 511, 483. 25043018 10.1038/nature13473 PMC6879323

[31] F. Wang , X. Wang , Y. Liu , Z. Zhang , Oxid. Med. Cell. Longevity 2021, 2021, 3846122. 10.1155/2021/3846122 PMC8500766 34630848

[32] A. Z. Jamurtas , Antioxidants 2018, 7, 50.29597252

[33] H. Sies , D. P. Jones , Nat. Rev. Mol. Cell Biol. 2020, 21, 363. 32231263 10.1038/s41580-020-0230-3

[34] D. E. Clapham , Cell 2007, 131, 1047. 18083096 10.1016/j.cell.2007.11.028

[35] H. Y. Zou , L. Guo , B. Zhang , S. Chen , X. R. Wu , X. D. Liu , X.‐Y. Xu , B.‐Y. Li , S. Chen , N.‐J. Xu , S. Sun , J. Clin. Invest. 2022, 132, e149160. 35426376 10.1172/JCI149160 PMC9012292

[36] R. L. Cornea , S. J. Gruber , E. L. Lockamy , J. M. Muretta , D. Jin , J. Chen , R. Dahl , T. Bartfai , K. M. Zsebo , G. D. Gillispie , D. D. Thomas , J. Biomol. Screening 2013, 18, 97. 10.1177/1087057112456878 PMC3721969 22923787

[37] R. Dahl , Bioorg. Med. Chem. 2017, 25, 53. 27776889 10.1016/j.bmc.2016.10.008

[38] J. A. Hawley , M. Hargreaves , M. J. Joyner , J. R. Zierath , Cell 2014, 159, 738. 25417152 10.1016/j.cell.2014.10.029

[39] D. Dutta , R. K. Paidi , S. Raha , A. Roy , S. Chandra , K. Pahan , Cell Rep. 2022, 40, 111058. 35830804 10.1016/j.celrep.2022.111058 PMC9308946

[40] K. Contrepois , S. Wu , K. J. Moneghetti , D. Hornburg , S. Ahadi , M.‐S. Tsai , A. A. Metwally , E. Wei , B. Lee‐McMullen , J. V. Quijada , S. Chen , J. W. Christle , M. Ellenberger , B. Balliu , S. Taylor , M. G. Durrant , D. A. Knowles , H. Choudhry , M. Ashland , A. Bahmani , B. Enslen , M. Amsallem , Y. Kobayashi , M. Avina , D. Perelman , S. M. Schüssler‐Fiorenza Rose , W. Zhou , E. A. Ashley , S. B. Montgomery , H. Chaib , et al., Cell 2020, 181, 1112. 32470399 10.1016/j.cell.2020.04.043 PMC7299174

[41] K. Dadson , L. Hauck , Z. Hao , D. Grothe , V. Rao , T. W. Mak , F. Billia , Sci. Rep. 2017, 7, 41490. 28148912 10.1038/srep41490 PMC5288653

[42] J. L. Braun , H. N. Messner , R. E. G. Cleverdon , R. W. Baranowski , S. I. Hamstra , M. S. Geromella , J. A. Stuart , V. A. Fajardo , Physiol. Rep. 2022, 10, 15285. 10.14814/phy2.15285 PMC9114654 35581738

[43] T. Ganz , J. Am. Soc. Nephrol. 2007, 18, 394. 17229910 10.1681/ASN.2006070802

[44] W. Xu , T. Barrientos , N. C. Andrews , Cell Metab. 2013, 17, 319. 23473029 10.1016/j.cmet.2013.02.004 PMC3594794

[45] E. Wyart , M. Y. Hsu , R. Sartori , E. Mina , V. Rausch , E. S. Pierobon , M. Mezzanotte , C. Pezzini , L. B. Bindels , A. Lauria , F. Penna , E. Hirsch , M. Martini , M. Mazzone , A. Roetto , S. Geninatti Crich , H. Prenen , M. Sandri , A. Menga , P. E. Porporato , EMBO Rep. 2022, 23, 53746. 10.15252/embr.202153746 PMC8982578 35199910

[46] F. M. Alves , K. Kysenius , M. K. Caldow , J. P. Hardee , P. J. Crouch , S. Ayton , A. I. Bush , G. S. Lynch , R. Koopman , J. Cachexia Sarcopenia Muscle 2021, 12, 476. 33665974 10.1002/jcsm.12685 PMC8061412

[47] Z. Hao , G. S. Duncan , Y.‐W. Su , W. Y. Li , J. Silvester , C. Hong , H. You , D. Brenner , C. Gorrini , J. Haight , A. Wakeham , A. You‐Ten , S. McCracken , A. Elia , Q. Li , J. Detmar , A. Jurisicova , E. Hobeika , M. Reth , Y. Sheng , P. A. Lang , P. S. Ohashi , Q. Zhong , X. Wang , T. W. Mak , J. Exp. Med. 2012, 209, 173. 22213803 10.1084/jem.20111363 PMC3260869

[48] Q. Guo , Z. Xu , D. Zhou , T. Fu , W. Wang , W. Sun , L. Xiao , L. Liu , C. Ding , Y. Yin , Z. Zhou , Z. Sun , Y. Zhu , W. Zhou , Y. Jia , J. Xue , Y. Chen , X.‐W. Chen , H.‐L. Piao , B. Lu , Z. Gan , Sci. Adv. 2022, 8, abo0340. 10.1126/sciadv.abo0340 PMC9328690 35895846

[49] H. Kim , X. Xue , J Vis Exp. 2020, 23, 10.

[50] L. W. Babcock , A. D. Hanna , N. H. Agha , S. L. Hamilton , Skeletal Muscle 2020, 10, 33. 33198807 10.1186/s13395-020-00250-5 PMC7667765

