# Supplementary material for: HUWE1 in Skeletal Muscle Prevents Muscle Fatigue via Maintaining Iron and Calcium Homeostasis
Source: Adv Sci (Weinh). 2025 Nov 20;13(4):e16719. doi: 10.1002/advs.202516719 (PMC12822411; doi:10.1002/advs.202516719)
Supplement: Supplementary file 1 — Supporting Information [file ADVS-13-e16719-s001.docx]

**HUWE1 in skeletal muscle prevents muscle fatigue**

**via maintaining iron and calcium homeostasis**

Huike Jiao^1, *^, Yuting Du^2, *^, Danxia Zhou^3^, Meng Bai^2^, Xu Gao^2^, Zhenyue Hao^4^, Nan-Jie Xu^5^, Jiaqiang Liu^6^, Ying Huang^2^, Zhenji Gan^3^, Jing Zhang^1, +^

*^1^Institute for Translational Medicine on Cell Fate and Disease, Shanghai Ninth People's Hospital, Key Laboratory of Cell Differentiation and Apoptosis of National Ministry of Education, Department of Pathophysiology, Shanghai Jiao Tong University School of Medicine, 200025 Shanghai, China；*

*^2^Key Laboratory of Cell Differentiation and Apoptosis of National Ministry of Education, Department of Pathophysiology, Shanghai Jiao Tong University School of Medicine, 200025 Shanghai, China；*

*^3^The State Key Laboratory of Pharmaceutical Biotechnology and MOE Key Laboratory of Model Animal for Disease Study, Model Animal Research Center, Nanjing University Medical School, Nanjing University, 210000 Nanjing, China.*

^4^ *Princess Margaret Cancer Centre, University Health Network, Toronto, ON M5G 2C1, Canada.*

*^5^*[*Songjiang Hospital*](http://english.songjiang.gov.cn/living/healthcare/270.shtml) *and Songjiang Research Institute, Shanghai Key Laboratory of Emotions and Affective Disorders, Shanghai Jiao Tong University School of Medicine, 201600 Shanghai, China.*

*^6^Department of Oral and Cranio-maxillofacial Surgery, Shanghai Ninth People’s Hospital, Shanghai Jiao Tong University School of Medicine, 200011 Shanghai, China.*

*^*^These authors contributed equally to the work.*

*^+^To whom correspondence should be addressed.*

*E-mail:* [jingzhang@shsmu.edu.cn](mailto:jingzhang@shsmu.edu.cn) (Jing Zhang)

***Keywords:*** HUWE1*, Exercise, Iron overload, SERCA, Oxidative stress*

**Supplementary Figures and Figure legends**

**Supplementary Figure S1
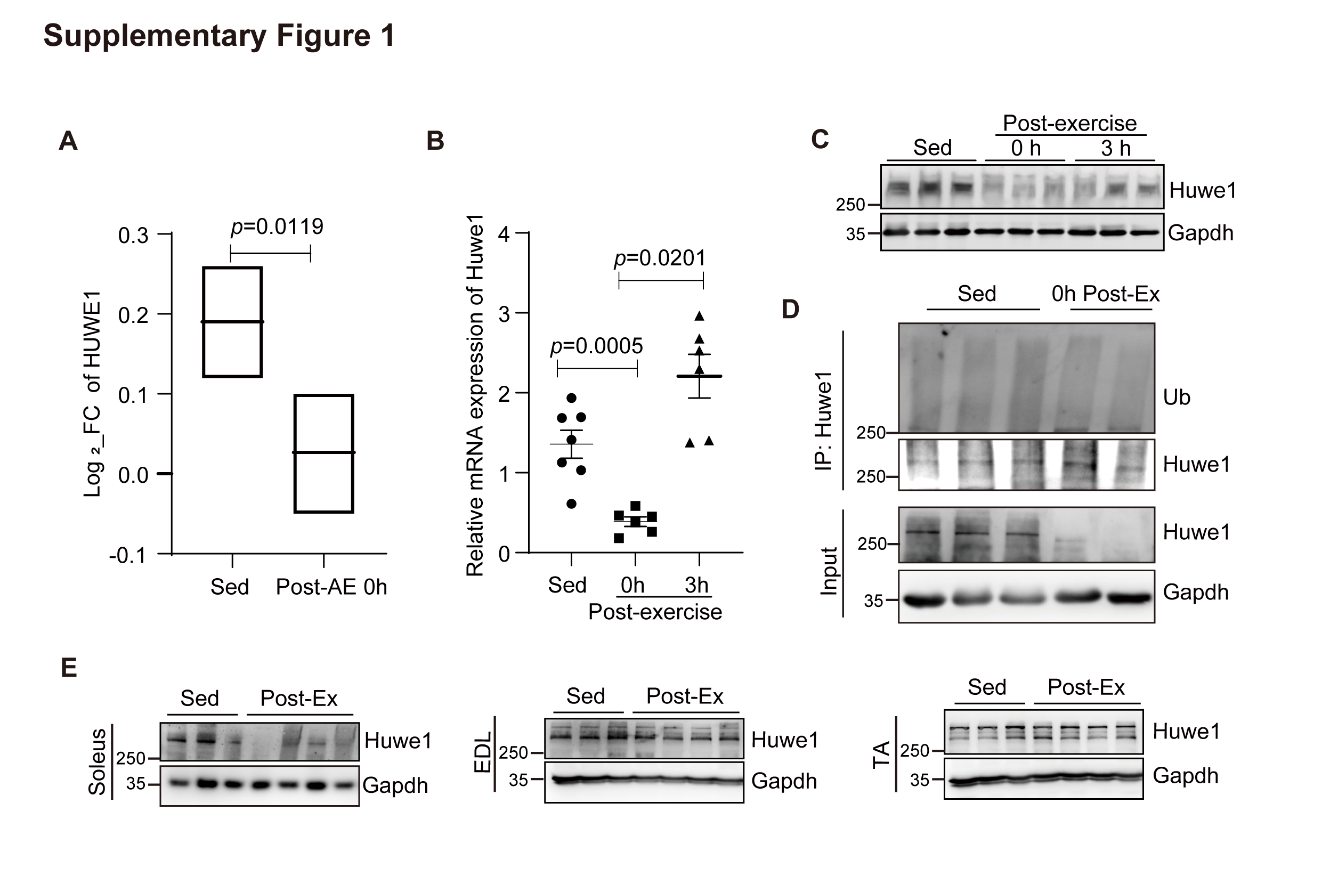
**

***Supplementary Figure S1. HUWE1 expression is suppressed upon exhausted exercise in soleus muscle.*** (A) HUWE1 expression was evaluated in human transcriptome under sedentary (Sed) and 0-hour post one bout of acute exercise (AE); (B-C) Male twelve-week-old C57BL/6 wild-type (WT) mice were subjected to one bout of exhausted exercise on multilane treadmill. Soleus muscles were collected from sedentary and exercise mice after indicated times (0 and 3 hours), and processed to detect the expression of Huwe1 by quantitative real-time PCR (qPCR, n=6, B) and western blotting (n=3, C); (D) Soleus muscles collected from mice at sedentary or 0 hours post-exhausted exercise were processed to Huwe1 immunoprecipitation, and then ubiquitination of Huwe1 was immunoblotted, n=2-3; (E) Male twelve-week-old C57BL/6 mice were subjected to one bout of exhausted exercise. Soleus, extensor digitorum longus (EDL) and tibialis anterior (TA) were collected from mice at sedentary (n=3) or subjected to exercise (n=4), and lysed for immunoblotting to detect the expression of Huwe1. Data are presented as Mean ± SEM. Student’s t-test was used to calculate the statistical probability (*p*) values shown between the indicated groups.

**Supplementary Figure S2**

***
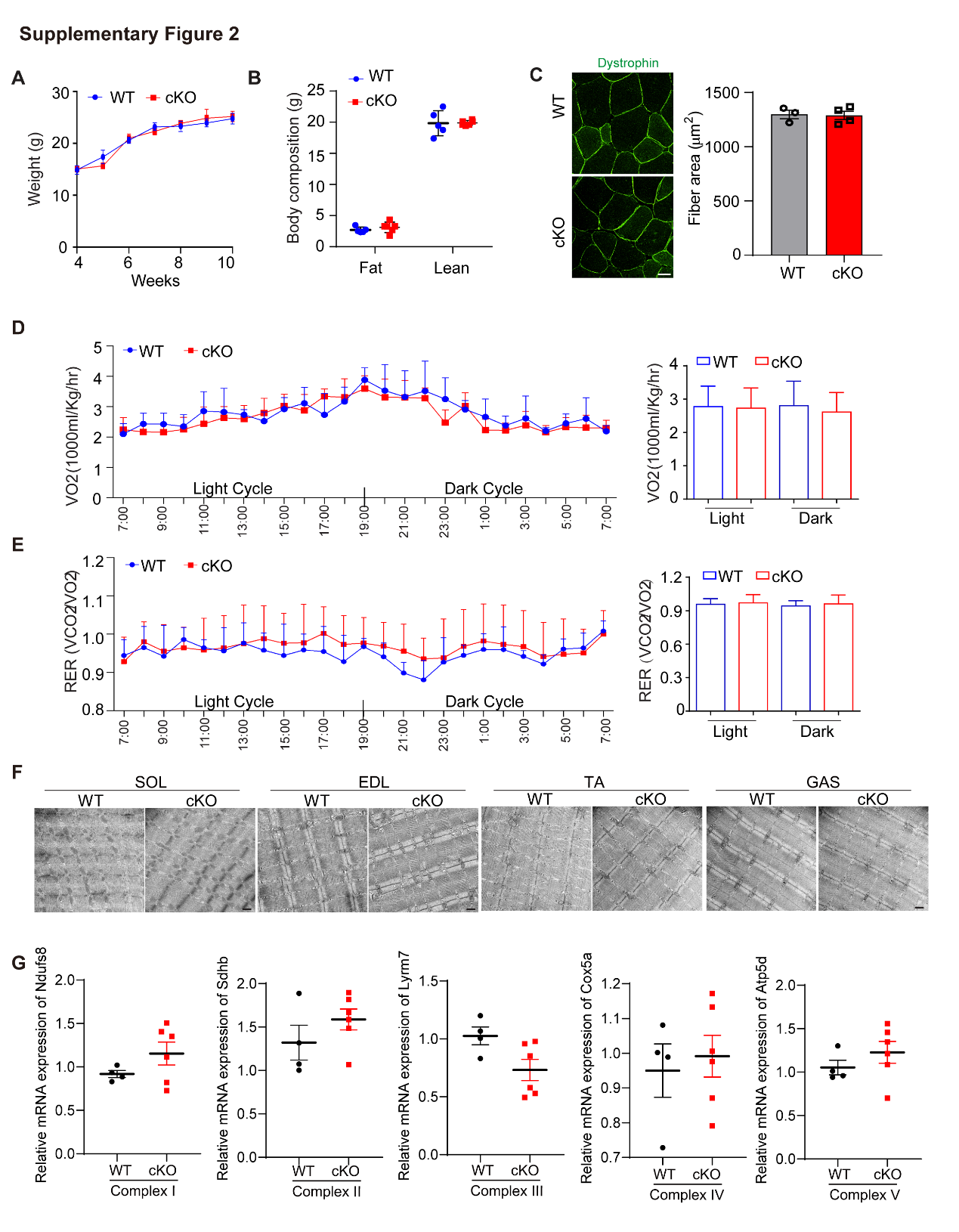
Supplementary Figure S2. Ablation of Huwe1 doesn’t influence body weight, body composition, fiber size of soleus muscles, morphology of mitochondrial and mice metabolism.*** (A) The body weight of WT and *Huwe1* cKO mice on chow diet were monitored at indicated times (n=5). (B) The body compositions of WT and *Huwe1* cKO mice at the age of 8 weeks (n=5). (C) Immunostaining of dystrophin in soleus sections of WT and *Huwe1* cKO mice. Quantified average fiber areas were shown as means ± SEM, n=3-4; Scale bar, 20 μm. (D-E) The oxygen consumption (VO_2_, D) and respiratory exchange ratio (RER, E) during the light and dark cycles in male WT and *Huwe1* cKO mice at the age of 12 weeks (n=8). (F) Representative TEM images of soleus (SOL), extensor digitorum longus (EDL), tibialis anterior (TA) and gastrocnemius (GAS) muscles in WT and *Huwe1* cKO mice; Scale bar, 500 nm. (G) mRNA expression of genes involved in the electron transport chain (ETC) was measured by qPCR in soleus muscle from WT and *Huwe1* cKO mice (n=4-6). Data are presented as Mean ± SEM. Student’s t-test was used to calculate the statistical probability (*p*) values shown between the indicated groups.

**Supplementary Figure S3**


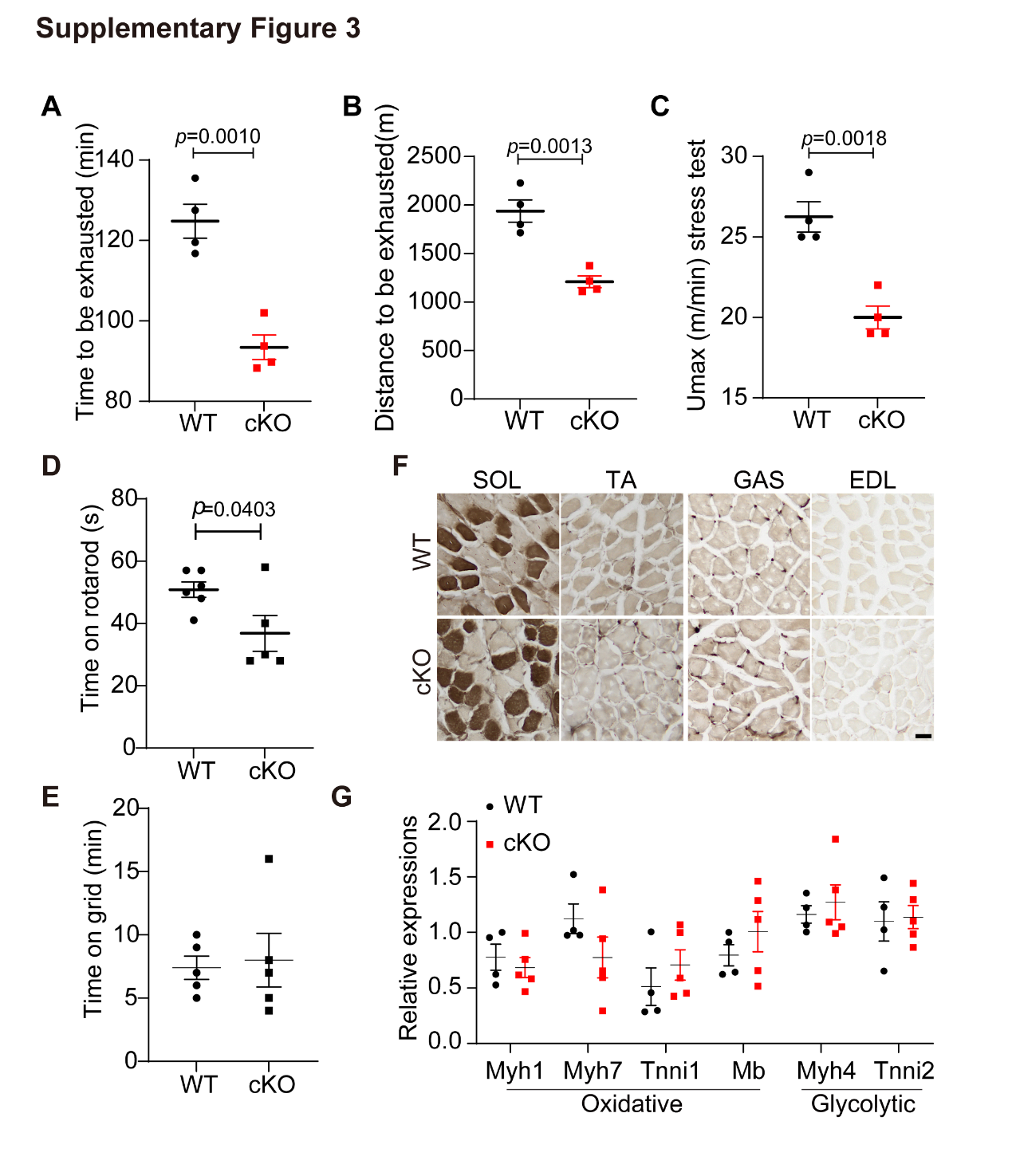


***Supplementary Figure S3. Mice with loss-of-function of Huwe1 in skeletal muscle display reduced exercise capacity.*** (A-C) WT and *Huwe1* cKO female mice were subjected to one bout of exhausted exercise (n=4). Running time (A), running distance (B) and maximum speed (C) of each mouse were recorded. (D-E) The time of WT and *Huwe1* cKO mice on rotarod (n=5-6, D) and grid (n=5, E). (F) ATPase staining (pH=4.3) of soleus (SOL), tibialis anterior (TA), gastrocnemius (GAS) and extensor digitorum longus (EDL) from both WT and *Huwe1* cKO mice. Scale bar, 50 μm. (G) mRNA expression of both oxidative and glycolytic muscle fiber-type markers was measured by qPCR in soleus muscle from WT and *Huwe1* cKO mice (n=4-5). Data are presented as Mean ± SEM. Student’s t-test was used to calculate the statistical probability (*p*) values shown between the indicated groups.

**Supplementary Figure S4**


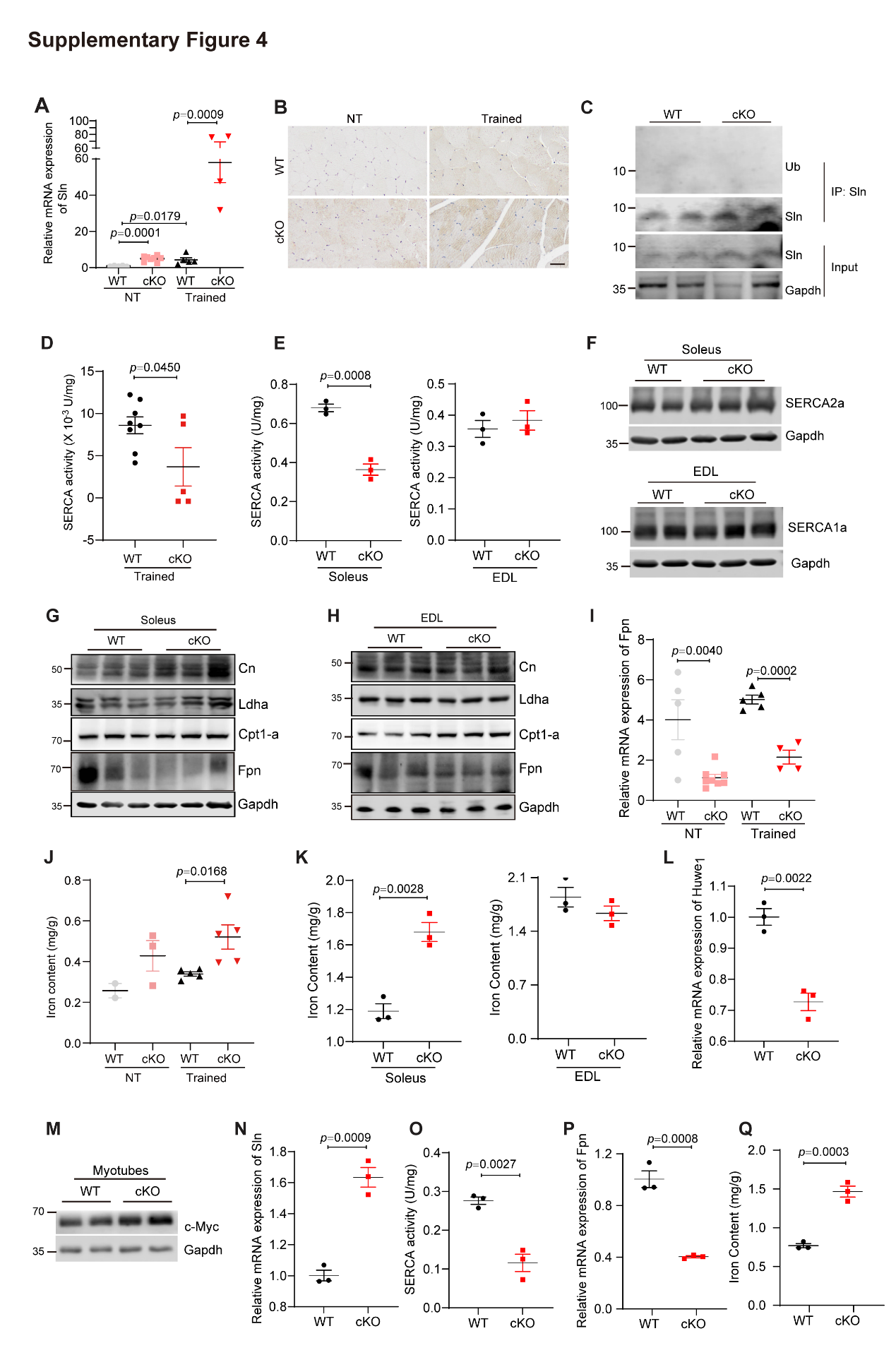


***Supplementary Figure S4. Sln is upregulated and Fpn is downregulated in skeletal muscle of Huwe1 deficient mice, leading to blunted SERCA activity and accumulated iron contents.*** (A-B) WT and *Huwe1* cKO mice were subjected to 6-week endurance training (Trained) or untrained (Not trained, NT, n=4-8). Soleus muscles were collected and processed to assess the mRNA expression of Sln by qPCR (A); Immunohistochemistry staining against Sln in soleus muscles from WT and *Huwe1* cKO mice subjected to endurance training or untrained, scale bar, 100 μm; Hematoxylin staining indicated the nuclei (B); (C) The soleus muscles of WT and *Huwe1* cKO mice were collected, and lysed for immunoprecipitation using Sln antibody, followed by western blotting against ubiquitin and Sln antibodies (n=2). (D) SERCA activity was detected in WT and *Huwe1* cKO mice post 6-week endurance training (n=5-8); (E) SERCA activity of the soleus muscle and EDL was measured in WT and *Huwe1* cKO mice (n=3); (F) The protein levels of SERCA2a/SERCA1a in the soleus muscle/EDL of WT and *Huwe1* cKO mice was detected by western blotting using specific antibody against SERCA2a and SERCA1a respectively (n=2-3); (G-H) The protein level of calcineurin (Cn), lactate dehydrogenase (Ldha), carnitine palmitoyl transferase-1 (Cpt1-a) and Fpn in the soleus muscle (G) and EDL (H) of WT and *Huwe1* cKO mice was detected by western blotting using specific antibodies (n=3); (I-J) The mRNA expression of Fpn (I) and the iron content (J) in soleus muscles of WT and *Huwe1* cKO mice subjected to endurance training or untrained (n=4-5); (K) The iron content in soleus muscles and EDL of WT and *Huwe1* cKO mice (n=3). (L-Q) The primary myoblasts were isolated from the soleus muscle of WT and *Huwe1* cKO mice, and then differentiated into myotubes (n=2-3 in each group). The RNA level of Huwe1 (L) and the protein expression of c-Myc (M) were detected by quantitative real-time PCR and western blotting respectively. The mRNA expression of Sln was assessed by qPCR (N); SERCA activity was detected (O); The mRNA expression of Fpn (P) and the iron content (Q) were measured. Data were presented as Mean ± SEM. Student’s t-test was used to calculate the statistical probability (*p*) values shown between the indicated groups.

**Supplementary Figure S5**

**
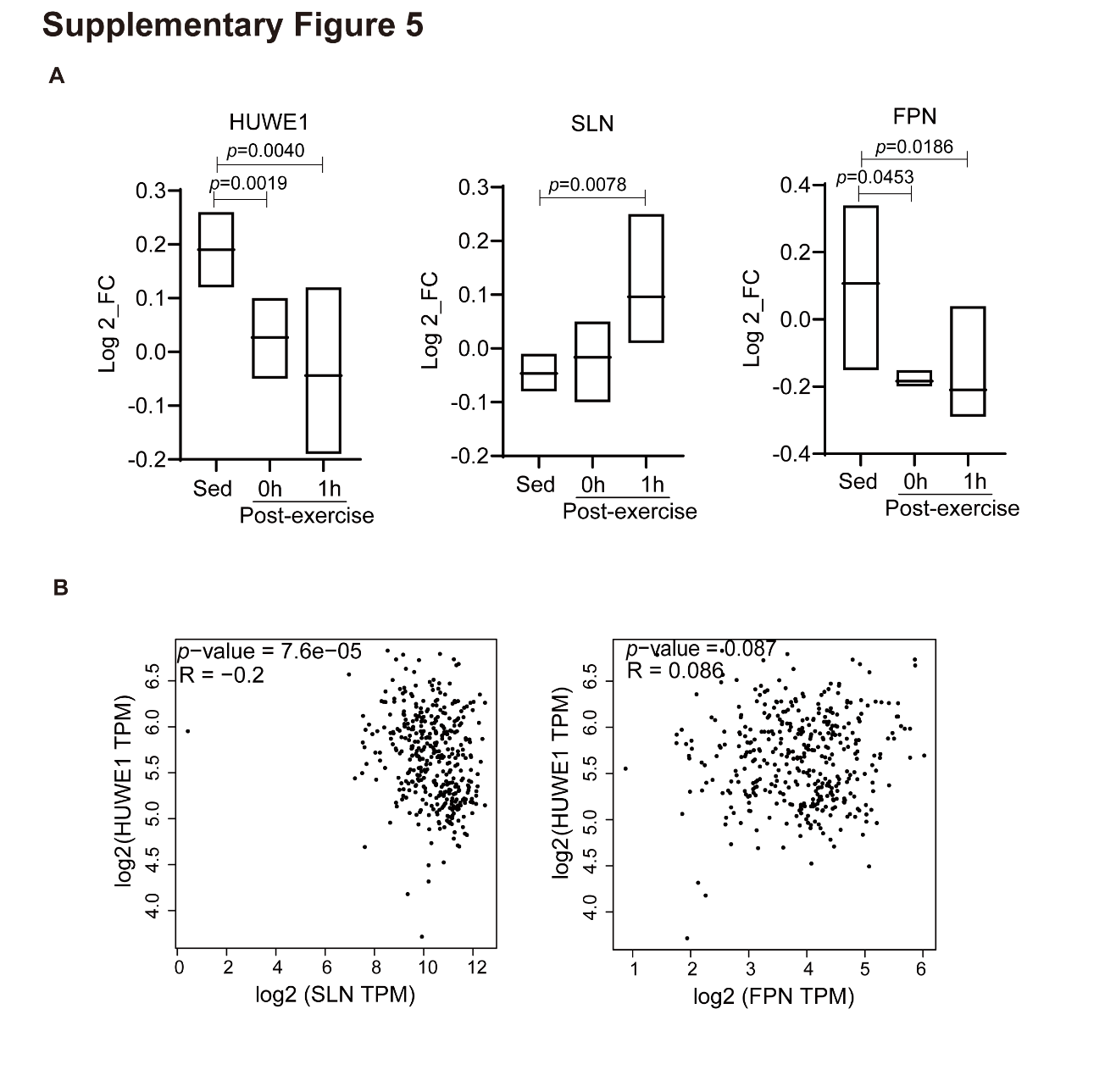
**

***Supplementary Figure S5. SLN is negatively correlated and FPN is potentially positive correlated with HUWE1 in human skeletal muscles.*** (A) Expression of HUWE1, SLN and FPN in 27 transcriptomic datasets from skeletal muscles of healthy humans after acute aerobic exercise (0 or 3 hours), compared to those at sedentary. (B) Correlation of expressions between HUWE1 and SLN (R=-0.2, *p*=7.6 X 10^-5^), or HUWE1 and FPN (R=0.086, *p*=0.087) in the database of human skeletal muscle in GTEx, statistically analyzed by Pearson Correlation Coefficient method.

**Supplementary Figure S6**


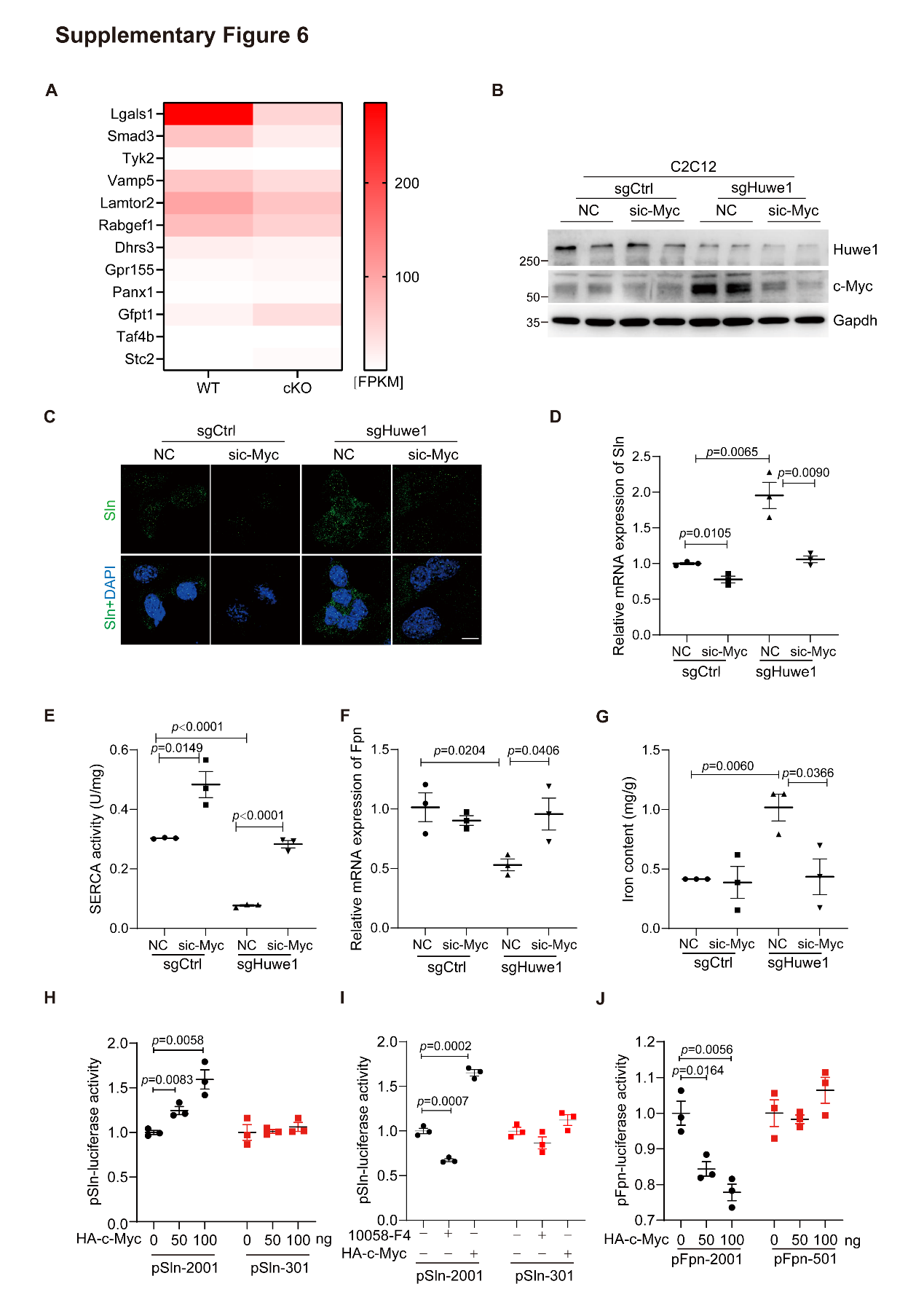


***Supplementary Figure S6. Silence of Huwe1 modulates Sln and Fpn expression via stabilizing c-Myc protein.*** (A) Heat map exhibiting the impact of Huwe1 deficiency in soleus muscles on previously validated c-myc target genes which demonstrated that myc activation was a consequence of Huwe1 deletion. (B-G) WT and Huwe1 KO C2C12 cells were transfected with siRNA against c-Myc for 48 hours. n=2-3 in each group as indicated. Expression of Huwe1 and c-Myc was detected by immunoblotting, Gapdh served as an internal control (B); The expression of Sln was assessed by immunofluorescence (Scale bar, 10 μm, C) and qPCR (D); SERCA activity was evaluated (E); The mRNA level of Fpn was measured by qPCR (F), and the iron content was determined (G). (H-I) HEK293T cells were transiently transfected with luciferase reporter construct pSln-2001 (with the sequence from TSS to -2001bp) or pSln-301 (with the sequence from TSS to -301bp) and co-transfected with increasing amount of HA-tagged c-Myc (H) or treated with 10058-F4 (60 μM for 24 hours, I), then luciferase activity was measured (n=3 in each group). (J) HEK293T cells were transiently transfected with luciferase reporter construct pFpn-2001 (with the sequence from TSS to -2001bp) or pFpn-501 (with the sequence from TSS to -501bp), and co-transfected with increasing amount of HA-tagged c-Myc, then luciferase activity was measured (n=3 in each group). Data were presented as Mean ± SEM. Student’s t-test was used to calculate the statistical probability (*p*) values shown between the indicated groups.

**Supplementary Figure S7**

**
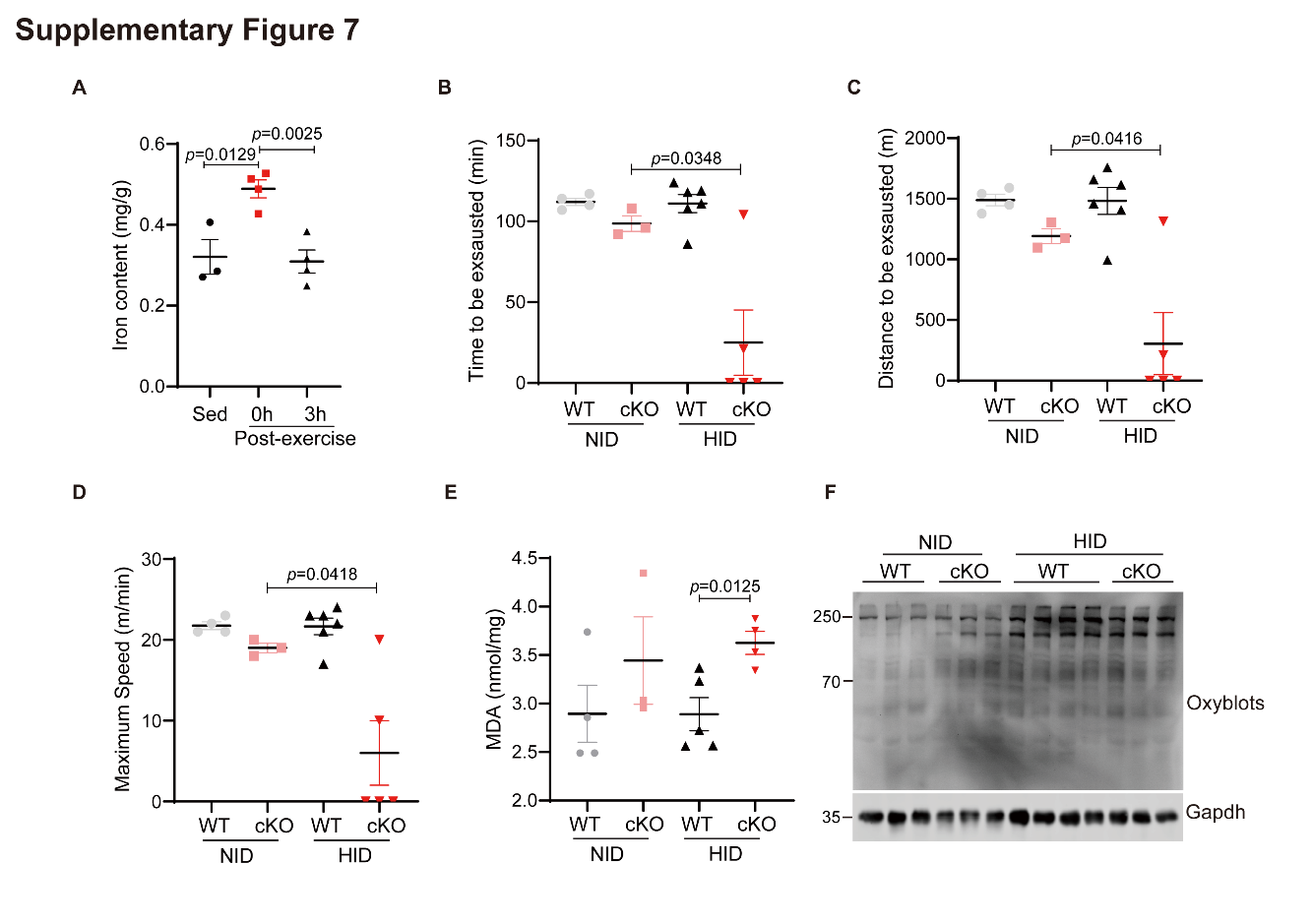
**

***Supplementary Figure S7.*** ***High-iron diet leads to dramatic loss of exercise performance in Huwe1 cKO mice.*** (A) Twelve-week-old C57BL/6 wild-type (WT) mice were subjected to one bout of exhausted exercise on multilane treadmill. Iron content in soleus muscles of mice at sedentary and mice with exhausted exercise after indicated time were detected, n=3-4 in each group. (B-D) WT and *Huwe1* cKO mice were fed with control diet (NID, 37mg carbonyl iron/kg) or high iron diet (HID, 8.3g carbonyl iron/kg) for 8 weeks, then subjected to one bout of exhausted exercise. Running time (B), running distance (C) and maximum speed (D) of each mouse were recorded, n=3-6 in each group; (E-F) MDA content (E) and immunoblots of protein carboxylation (F) in soleus muscles of WT and *Huwe1* cKO mice fed with control diet (NID) or high iron diet (HID) were evaluated, n=3-5 in each group as indicated. Data were presented as Mean ± SEM. Student’s t-test was used to calculate the statistical probability (*p*) values shown between the indicated groups.

**SUPPLEMENTARY MATERIALS AND METHODS**

**Grip strength Measurement**

The screen is a 43 cm square of wire mesh consisting of 12 mm squares of 1 mm diameter wire. The mesh is surrounded by a 4 cm deep wooden beading. Before the strength measurement, prepare the mice to make sure they are properly awake. To start the measurement, place the mouse in the center of the screen and start a stop clock. Then quickly rotate the screen to an inverted position within 2 sec, with the head of mouse declining first. Hold the screen 40-50cm above a padded and surrounded ground. Record the time when the mouse was exhausted and falls off, or stop the test when 60 sec is reached.

**Immunohistochemistry/fluorescence Staining and Transmission Electron Microscopy imaging**

Fresh mice soleus muscles were fixed in 4% paraformaldehyde (PFA) overnight. Tissues were then embedded in paraffin and sectioned to 4μm-thick slices which were used for immunohistochemistry/fluorescence analysis. Soleus sections were rehydrated and processed for an antigen-retrieval procedure. For immunohistochemistry staining, soleus muscle sections were incubated with primary antibodies against Sln (1:100, 18395-1-AP, Proteintech) overnight at 4 °C, followed by biotinylated secondary antibodies (Vector Labs, CA, USA). Immunoreactive cells were observed with DAB (Vector Labs) under the microscope. For immunofluorescence staining, soleus sections were incubated with primary antibodies against Dystrophin (1:100, Ab15277, Abcam) overnight at 4 °C followed by goat anti-mouse Alexa Fluor 647 and goat anti-rabbit Alexa Fluor 488 secondary antibody (Life Technologies). Tissues were imaged by an FV3000 Laser Scanning Confocal Microscope (Olympus, Tokyo, Japan). Transmission electron microscopy was performed using a Tecnai 10 Electron Microscope (FEI, Hillsboro, OR) at the Electron Microscopy Core Facility, Shanghai Jiao Tong University School of Medicine. For each section, three to six images from random fields were taken, and at least three mice per group were subjected to each experiment.

**Mice Metabolic Studies**

Mice from both genotypes were acclimated to Tecniplast E-chiller metabolic cages (Tecniplast, Italy) with access to sufficient water and food, undergoing a 24 h adaptation period. Following this acclimation, carbon dioxide production (VCO_2_) and oxygen consumption (VO_2_) were continuously monitored over 24 h for 3 days. VO_2_ and VCO_2_ levels were monitored at 1h intervals for each mouse. The analysis involved calculating the Respiratory Exchange Rate (RER), determined as the ratio of VCO_2_ to VO_2_ (VCO_2_/VO_2_). VO_2_ were normalized to body weight. Fat mass and lean mass were assessed by a Body Composition Analyzers for Live Small Animals and Organs (Echo-MRI 100H, Houston, USA).

**Histology and ATPase staining**

Soleus (SOL), tibialis anterior (TA), gastrocnemius (GAS) and extensor digitorum longus (EDL) from both WT and *Huwe1* cKO mice were dissected and frozen in optimal cutting temperature (OCT) medium using liquid nitrogen-cooled isopentane. Fresh muscle samples were sectioned at 10-μm thickness, mounted on charged glass slides, and used for Hematoxylin and Eosin (H&E) and ATPase staining. ATPase staining was done by using ATPase staining kit (Gefanbio, China) following the manufactory`s instruction. Briefly, incubate each muscle sample in the pre-incubation solution with pH at 4.3 for 10 minutes. After the pre-incubation solution were poured out, muscle samples were incubated in ATP solution for 30 minutes at 37°C, followed with 2% cobalt chloride solution for 3 minutes. Samples were fully washed under the tap water and then rinsed with deionized water. Then ammonium sulfide solution worked for 1-3 minutes until cobalt was precipitated as a black insoluble compound. Dehydrate slides in ascending alcohols (50%, 70%, 80%, 95%, 100%) and clear with at least two changes of xylene before mounting.

**Western Blot**

Muscle proteins were extracted with RIPA buffer (50 mM Tris-HCl with pH 7.4; 150 mM NaCl; 0.1% SDS; 2 mM EDTA; 50 mM NaF; 0.5% Deoxycholate and 1% NP-40), containing Protease Inhibitor Cocktail (APExBIO, Houston, TX, USA). C2C12 and HEK293T protein samples were extracted with laemmli buffer (50 mM Tris-HCl with pH 7.5; 2% SDS; 2 mM EDTA; 20 mM NaF). For Immunoprecipitation of c-Myc or Huwe1 in muscles, soleus samples were lysed in TAP buffer (20mM Tris-HCl, pH 7.5; 150 mM NaCl; 1mM NaF;1mM Na_3_VO_4_; 1mM EDTA and 0.5%NP-40). Protein concentration was quantified by using BCA Protein Assay Kit (Thermo Fisher Scientific, Waltham, MA, USA). 30 µg of protein lysates were subjected to Tris-SDS-PAGE. 500µg proteins were subjected for immunoprecipitation. For small molecular protein Sln, protein lysate was separated by using Tricine-SDS-PAGE. Immunoblot analysis was performed by using antibodies specific to HUWE1/Lasu1 (Bethyl Laboratories, Montgomery, USA), Fpn/Slc40a1 (Alpha Diagnostic International), c-Myc (Santa Cruz Biotechnology), Tfr1 (Abclonal), Tfr2 (Signalway Antibody), Flag (Sigma-Aldrich), Ub (Abclonal) and GAPDH (Proteintech). Protein oxidation in soleus skeletal muscle and C2C12 myoblasts were assessed by immunoblotting using the OxyBlot Protein Oxidation Detection Kit according to the manufacturer’s instructions (Merck Millipore, Burlington, MA).

**Luciferase reporter assay**

Cells were transiently transfected with constructs expressing Renilla luciferase under a Sln promoter (-2000bp) or Fpn (-2000bp) promoter with an SV40-firefly luciferase construct (pProUTR-reporter) together with empty control vector or HA-tagged c-Myc. Cells were cultured for 36 hours and assayed for luciferase activity with the Dual Luciferase Reporter Gene Assay Kit (Yeasen). Or 24 hours after transient transfection, cells were treated with c-Myc inhibitor 10058-F4 (60 μM) for additional 24 hours and then signed for assay of luciferase activity.

**Primary myoblast isolation, culture, and differentiation**

Primary skeletal muscle myoblasts were isolated from 6 to 8-week-old mice as previously described^1^. Briefly, hindlimb skeletal muscles were rinsed with phosphate-buffered saline. Samples were minced with scissors and transferred to 10 ml of collagenase type II (400U/ml; Worthington) and Dispase II (1 U/ml; Roche, Germany) in DMEM at 37°C with shaking for 1 hour. The cell suspension was dispersed 20 times with a 10-ml pipette and centrifuged at 1400g for 5 minutes. The cell pellet was resuspended in DMEM with 10% FBS and filtered through 70-µm and then 40-µm cell strainers (BIOFIL, China). After centrifugation at 1400g for 5 minutes, cell pellet was resuspended in Ham’s F10 with 20% FBS and bFGF (10 ng/ml). Cell suspension was then plated on collagen-coated culture dishes. After 5 times pre-plating to purifying myoblast to > 98%, myoblast cells were plated into collagen-coated 6-well plates. Medium was changed to DMEM with 4% horse serum (VivaCell) without bFGF to induce differentiation for 7 days prior to experiments.

**Chromatin immunoprecipitation**

Chromatin immunoprecipitation was performed as previously described^2^. Briefly, two 15-cm dishes of 85% confluent C2C12 cells with empty control or Flag-tagged c-Myc were cross-linked and harvested. Cells were lysed on ice with 1 ml ChIP lysis buffer. DNA was sheared by sonication (30-second on/30-second off, 18 cycles; Active Motif). To determine DNA concentration, 20 µl sheared lysate was tested and purified using Chromatin IP DNA Purification Kit (Active Motif). For ChIP, lysate volumes corresponding to 40 μg DNA were diluted in dilution buffer and pre-cleared by Normal mouse IgG antibody (SantaCruz Biotechnology) and protein A/G Sepharose mixture. Subsequently, 1 μg Flag (Sigma) or normal mouse IgG antibodies were added and the samples were rotated at 4 °C overnight. Protein A/G Sepharose mixture were added for additional incubation at 4°C for 3 hours. Beads were collected and washed once in low salt buffer, once in high salt buffer, once in LiCl buffer and twice in TE buffer for each 5 minutes on a rotator at 4 °C. Beads were finally resuspended in 100 μl TE buffer. In parallel, 1/10 volume (4μg) of the initial lysate (10% input samples) was also diluted with 100 μl TE buffer. After 30 minutes incubation at 37 °C with RNase A, samples were incubated with proteinase K for 2 hours at 37 °C followed by incubation at 65 °C overnight. Then, samples were purified and DNA was eluted for quantification by real-time PCR. Each reaction mixture contained 2 μl of ChIP DNA or 1% input DNA. The ChIP-PCR primers for the Sln and Fpn in the promoter are listed in **Supplementary Table S2**. Calculation of enrichment by immunoprecipitation relative to the signals obtained for 1% input DNA was performed according to the following equation: percent of (input)=2^-(Ct sample-Ct input)^_._

**Supplementary Table S1**

**Real-time PCR primers**

| **Genes** | **Forward Sequence 5’-3’** | **Reverse Sequence 5’-3’** |
| --- | --- | --- |
| Huwe1 | TCTTCCACTAGAGATTCTGCCG | TGATACCAGCAAGGGGATCTTC |
| Sln | ATGGAGAGGTCTACTCAGGAGCTG | TCAGTATTGGTAGGACCTCACGAGG |
| Fpn | ACCAAGGCAAGAGATCAAACC | AGACACTGCAAAGTGCCACAT |
| c-myc | ATGCCCCTCAACGTGAACTTC | GTCGCAGATGAAATAGGGCTG |
| Sdh | GCTAAGGGCGAGAACCTGTC | CATGCTCCCAGTAGTGAACATC |
| Myh1 | CGGAGTCAGGTGAATACTCACG | GAGCATGAGCTAAGGCACTCT |
| Myh4 | AAACCACCTCAGAGTTGTGGA | GTTCCGAAGGTTCCTGATTGC |
| Myh7 | ACTGTCAACACTAAGAGGGTCA | TTGGATGATTTGATCTTCCAGGG |
| Tnni1 | ATGCCGGAAGTTGAGAGGAAA | TCCGAGAGGTAACGCACCTT |
| Tnni2 | AGAGTGTGATGCTCCAGATAGC | AGCAACGTCGATCTTCGCA |
| Mb | GGAAGTCCTCATCGGTCTGT | GGTCCTCTGAGCCCTTCATA |
| Ndufs8 | AGTGGCGGCAACGTACAAG | TCGAAAGAGGTAACTTAGGGTCA |
| Sdhb | AATTTGCCATTTACCGATGGGA | AGCATCCAACACCATAGGTCC |
| Lyrm7 | GTCAGCCCGCCAAGGTTTTA | CAGTACGGCACATTTTCTGTGA |
| Cox5a | GCCGCTGTCTGTTCCATTC | GCATCAATGTCTGGCTTGTTGAA |
| Atp5d | TGCTTCAGGCGCGTACATAC | CACTTGCTTGACGTTGGCA |
| Rplp0 | AGATGCAGCAGATCCGCAT | GTTCTTGCCCATCAGCACC |

**Supplementary Table S2**

**ChIP primers for real-time PCR**

| **Genes** | **Forward Sequence 5’-3’** | **Reverse Sequence 5’-3’** |
| --- | --- | --- |
| pSln-p1 | CGTGTGAATATGTGTATGGAC | GCAACGCTTTCAGTGTAACA |
| pSln-p2 | GGCTTACAATTGTCTGGGAC | GTCGAAACGAGTAGGAATGTG |
| pSln-p3 | CTCCACATTGCAAACAGCTAG | CTTTTGATGTAGTGGTGTGGG |
| pFpn-p1 | GTCAATTTCCCCTCTCACATG | CATCTTCAGTGTGTGCATGG |
| pFpn-p2 | CCAGGAGCTCAGGACAATG | GGACTCTCAGTTCAGTGCCC |

**Supplementary Table S3**

| **Products** | **Company** | **Catalog No.** |
| --- | --- | --- |
| **Antibodies** | | |
| Lasu1/Ureb1 Antibody | Bethyl Laboratories | A300-486A-T |
| SLN antibody | Proteintech | 18395-1-AP |
| FPN/SLC40A1 antibody | Proteintech | 26601-1-AP |
| FPN antibody | Alpha Diagnostic Intl. Inc. | MTP11-A |
| TfR1 Monoclonal Antibody | Abclonal | A5865 |
| TfR2 Polyclonal Antibody | Signalway Antibody | 31754 |
| c-Myc (A14) antibody | SantaCruz Biotechnology | sc-786 |
| c-Myc (N262) antibody | SantaCruz Biotechnology | sc-764 |
| c-Myc Antibody | Proteintech | 67447-1-Ig |
| Ub Antibody | Abclonal | A19686 |
| Dystrophin | Abcam | Ab15277 |
| Flag antibody | Sigma-Aldrich | F1804 |
| HA antibody | Sigma-Aldrich | H9658 |
| Normal mouse IgG | SantaCruz Biotechnology | sc-2025 |
| LDHA antibody | Abclonal | A1146 |
| CPT1-A antibody | AiFang | AFRM9449 |
| Calcineurin A antibody | Abclonal | A4346 |
| SERCA1/ ATP2A1 antibody | Abclonal | A19639 |
| SERCA2/ATP2A2 antibody | Abclonal | A11692 |
| GAPDH Monoclonal Antibody | Proteintech | 60004-1-Ig |
| **Chemicals and Diets** | | |
| 10058-F4 | Beyotime | SC6650 |
| Cycloheximide | MedChemExpress | HY-12320 |
| CDN1163 | Targetmol | 892711-75-0 |
| Ionomycin | Beyotime | S1672 |
| Protease Inhibitor Cocktail | APExBIO | K1007 |
| Normal-iron diet | Dyets | AIN-76A-Fe-37mg |
| Low-iron diet | Dyets | AIN-76A-Fe-18.5 mg |
| High-iron diet | Dyets | AIN-76A-Fe-8.3g |
| Lipofectamine RNAiMAX | Invitrogen | 13778-150 |
| PEI MAX | Polysciences | 24765-1 |
| On-Target plus Mouse Myc siRNAs | Dharmacon | L-040813-00-0005 |
| Collagenase type 2 | Worthington | LS004176 |
| Dispase II | Roche | 4942078001 |
| Horse serum | VivaCell | C2510 |
| **Critical Assays** | | |
| Ca^2+^-ATPase Assay Kit | NanjingJiancheng | A070-4-2 |
| Tissue Iron Assay Kit | NanJingJianCheng | A039-2-1 |
| OxyBlot Protein Oxidation Detection Kit | Merck | S7150 |
| GSH/GSSG-Glo Assay Kit | Promega | V6612 |
| MitoTracker™ Orange CMTMRos | Invitrogen | M7510 |
| ROS assay kit-highly sensitive DCFH-DA | Dojingo | R252 |
| FerroOrange | Dojingo | F374 |
| BCA Assay Kit | Thermo Fisher Scientific | 23235 |
| Fura-2 | Thermo Fisher Scientific | F1201 |
| TRIzol^TM^ Reagent | Invitrogen | 15596018CN |
| Superscript II reverse transcriptase Kit | Vazyme | R201-01 |
| BrightCycle Universal SYBR Green qPCR Mix with UDG | Abclonal | RK21219 |
| Genious 2X SYBR Green Fast qPCR Mix | Abclonal | RK21206 |
| Dual Luciferase Reporter Gene Assay Kit | Yeasen | 11402ES60 |
| ATPase staining kit | Gefanbio | M091 |
| Chromatin IP DNA Purification Kit | Active motif | 58002 |
| Tricine-SDS-PAGE Gel Preparation kit | Sangon Biotech | C641100-0150 |

**References**

1. L. Hindi, McMillan, J.D., Afroze, D., Hindi, S.M. & Kumar, A. Isolation, Culturing, and Differentiation of Primary Myoblasts from Skeletal Muscle of Adult Mice. *Bio Protoc* (2017) **7**.

2. H. Dopeso, Jiao, H.K., Cuesta, A.M. *et al.* PHD3 Controls Lung Cancer Metastasis and Resistance to EGFR Inhibitors through TGFα. *Cancer Res* (2018) **78**, 1805-1819.
